# Overexpression of UBQLN1 reduces neuropathology in the P497S UBQLN2 mouse model of ALS/FTD

**DOI:** 10.1186/s40478-020-01039-9

**Published:** 2020-10-07

**Authors:** Shaoteng Wang, Micaela Tatman, Mervyn J. Monteiro

**Affiliations:** Center for Biomedical Engineering and Technology and Department of Anatomy and Neurobiology, University of Maryland School of Medicine, Baltimore, MD USA

**Keywords:** Amyotrophic lateral sclerosis, UBQLN1, UBQLN2, Motor neuron disease, Proteostasis

## Abstract

Missense mutations in *UBQLN2* cause X-linked dominant inheritance of amyotrophic lateral sclerosis with frontotemporal dementia (ALS/FTD). UBQLN2 belongs to a family of four highly homologous proteins expressed in humans that play diverse roles in maintaining proteostasis, but whether one isoform can substitute for another is not known. Here, we tested whether overexpression of UBQLN1 can alleviate disease in the P497S UBQLN2 mouse model of ALS/FTD by crossing transgenic (Tg) mouse lines expressing the two proteins and characterizing the resulting genotypes using a battery of pathologic and behavioral tests. The pathologic findings revealed UBQLN1 overexpression dramatically reduced the burden of UBQLN2 inclusions, neuronal loss and disturbances in proteostasis in double Tg mice compared to single P497S Tg mice. The beneficial effects of UBQLN1 overexpression were primarily confirmed by behavioral improvements seen in rotarod performance and grip strength in male, but not female mice. Paradoxically, although UBQLN1 overexpression reduced pathologic signatures of disease in P497S Tg mice, female mice had larger percentage of body weight loss than males, and this correlated with a corresponding lack of behavioral improvements in the females. These findings lead us to speculate that methods to upregulate UBQLN1 expression may reduce pathogenicity caused by UBQLN2 mutations, but may also lead to gender-specific outcomes that will have to be carefully weighed with the therapeutic benefits of UBQLN1 upregulation.

**Electronic supplementary material:**

The online version of this article (10.1186/s40478-020-01039-9) contains supplementary material, which is available to authorized users.

## Introduction

**Fig. 1:**
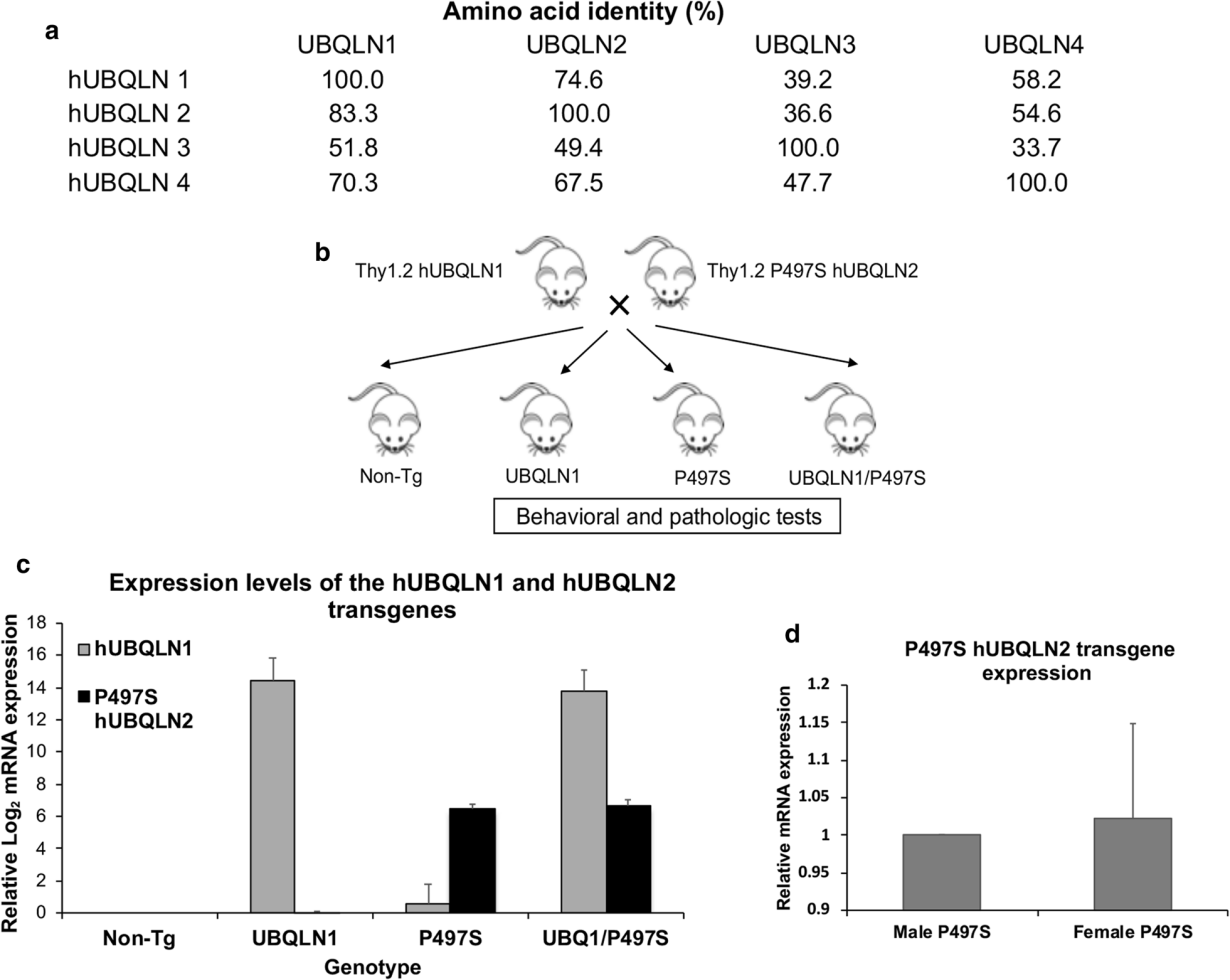
Sequence identity of human UBQLN isoforms and expression levels of WT hUBQLN1 and the P497S mutant hUBQLN2 transgenes in Tg mice. **a** Sequence identity of human UBQLN isoforms with one another. **b** Strategy of the mouse cross used to generate the four genotypes for the study. **c** Graph showing the relative mRNA expression levels of human UBQLN1 and the P497S mutant UBQLN2 transgenes in the brain of 24-week-old animals measured by RT qPCR analysis. **d** Comparison of P497S transgene mRNA expression by RT qPCR analysis of 24-week-old male and female mice

Ubiquilin (UBQLN) proteins function in regulation of proteostasis [15]. The proteins are highly conserved across species [19, 20]. Humans contain four UBQLN protein isoforms, encoded by separate genes that are differentially expressed in the body. UBQLN1 is the most widely expressed isoform, whereas UBQLN2 and UBQLN4 have more restricted expression patterns [4, 13, 19]. UBQLN3 is only expressed in the testis [3]. The proteins encoded by the four genes are all approximately 600 amino acids long. Of the four isoforms, UBQLN 1, 2 and 4 share the greatest homology (70–83% identity to one other), with UBQLN 3 being least related (~ 35%) (Fig. 1a). Interestingly, UBQLN1 and UBQLN2 are the most closely related isoforms (74 and 83%, respectively) (Fig. 1a). However, it is unclear whether the different UBQLN isoforms have unique or redundant functions.

Information published on the knockout (KO) of *UBQLN* genes in rodents indicates neither UBQLN1 nor 2 is essential for animal survival [17, 36]. The only deficiency noted was increased vulnerability to ischemic injury in UBQLN1 KO animals, suggesting UBQLN1 is neuroprotective [17]. This finding is consistent with transgenic studies showing overexpression of UBQLN1 can extend survival and is neuroprotective in cell and mouse models of Huntington’s and Alzheimer’s disease [1, 26, 32].

The lack of any overt phenotype in either UBQLN1 or UBQLN2 KO animals indicates the two proteins are dispensable for survival, although it remains unclear if this is due to compensation by other *UBQLN* genes as information on the survival of double KO mice is not available. Moreover, there is no study that shows one UBQLN isoform can substitute for another in animals. This knowledge may be especially useful in diseases linked to mutation of a particular *UBQLN* gene, where dysfunction in one could potentially be rescued by expression of another isoform. We became interested in this idea because of its therapeutic potential for treating diseases linked to *UBQLN2* mutations such as ALS/FTD. With this goal in mind we describe here the consequences of forced expression of UBQLN1 in the P497S UBQLN2 mouse model of ALS/FTD [14].

The P497S mutation is one of several missense mutations in *UBQLN2* that are linked to development of amyotrophic lateral sclerosis (ALS) alone or together with frontotemporal dementia (FTD) [5, 10]. Most UBQLN2 mutations map in a PXX repeat motif that is unique to the protein. The role of the repeat is not known. Several rodent models expressing different UBQLN2 mutations have been developed [2, 7, 11, 14, 23, 29, 36]. We selected the P497S UBQLN2 transgenic (Tg) mouse model for our investigations because it recapitulates central features of the human disease, including motor neuron (MN) disease [14]. We crossed the mutant line with Tg mice overexpressing wild type (WT) UBQLN1 [26] to determine whether UBQLN1 overexpression can alleviate disease. We present the behavioral and pathological studies of mice from the cross, showing that double transgenic mice have greatly reduced UBQLN2 pathology. However, the behavioral studies suggest that the rescue may be gender-specific, with better protection in males and possibly detrimental effects in females.

## Materials and methods

### Animal research

All animal procedures were approved by University of Maryland Baltimore Institutional Animal Care and Use Committee and conducted in full compliance as recommended in the NIH Guide for the Care and Use of Laboratory Animals.

### Mouse husbandry, breeding and genotyping

The generation of the transgenic mouse lines with Thy1.2 promoter driven expression of either full-length human wild type UBQLN1 or the ALS/FTD P497S UBQLN2 mutant proteins were described previously [14, 26]. The two mouse lines were backcrossed with C57BL/6J mice (The Jackson Laboratory, Strain 000664) for at least 10 generations prior to being crossed with one another for the experiments. Progeny from the UBQLN1 and UBQLN2 crosses were genotyped by polymerase chain reaction (PCR) analysis of isolated mouse tail DNA using a common 5′ primer (TCTGAGTGGCAAAGGACCTTA) with either a 3′ UBQLN1 (GGCAGTGCCGCTGGAAGCTGATGA) or a 3′ UBQLN2 (CGACGCCGAGGTAGTGTTAGTTCCC) primer, designed to specifically detect transmission of the human UBQLN1 or 2 transgenes, respectively. The resulting four genotypes (Non-Tg, single UBQLN1 Tg, single P497S UBQLN2 Tg, and UBQLN1:P497S UBQLN2 double Tg) were obtained in the expected Mendelian ratio. The mice were segregated by sex, but not genotype, and housed at no more than 5 animals per cage. The mice were fed regular mouse chow and given clean water to drink ad lib. Animals that had difficulty in reaching the food were provided with Diet-Gel 76A (Clear H_2_O, Portland, ME) in the bedding.

### Mouse behavioral studies

All tests were conducted by investigators who were blinded to the animal genotypes. Body weight was measured bi-weekly from 6 to 52 weeks of age to the nearest 0.1 g. The mean weight of at least 5 animals for each gender and genotype is shown.

Rotarod tests were conducted of animals from 6 till 52 weeks of age using a home-made accelerating rotarod apparatus. Mice were initially trained for 3 consecutive days at 5 weeks of age and then tested bi-weekly [14]. The latency to fall in the longer of two consecutive trials was used for analysis. The mean time of the latency to fall for at least 5 animals for each gender and genotype is shown.

Hind-limb grip strength was measured coincident with the rotarod tests using a grip strength meter [14]. The mean grip strength of at least 5 animals for each sex and genotype is presented.

### Mouse tissue isolation and cryosectioning

Animals were euthanized either by isoflurane or following intracardiac perfusion with 4% paraformaldehyde after reaching surgical plane of anesthesia when tissue preservation was required.

Spinal cords (SCs) and brains were dissected from euthanized mice and immediately frozen on dry ice and then transferred to − 80 °C for storage. Prior to cryosectioning the tissues were quickly thawed, immediately placed in ice cold 4% paraformaldehyde (PFA) in 1 × PBS followed by equilibration in 25% sucrose in 1 x PBS, and finally embedded in O.T.C. compound (Sakura Finetek USA, Inc., Torrance CA) [14]. Sagittal or coronal sections of the frozen brain and transverse sections of the SC (10–15 μm thick) were cut using a Leica CM 3050S cryostat (Leica Biosystems, Buffalo Grove, IL), numbered, and stored on slides at − 20 °C.

### Immunofluorescence staining of tissue sections

The procedure for immunofluorescent staining of brain and SC sections was described previously [26, 30]. The sections were stained with primary antibodies against the following proteins: mouse anti-UBQLN2 (#NBP2-25164, Novus Biologicals, Littleton, CO) or affinity-purified rabbit anti-UBQLN2 (UMY75 [26]), rabbit anti-TDP-43 (UMY112, rabbit antibody against the C-terminal region of TDP-43 (NQAFGSGNNSYSGS), produced in house), goat anti-ChAT (#AB144P Millipore-Sigma, Burlington, MA), mouse anti-SQSTM1/p62 (ab56416, Abcam, Eugene, OR), rabbit anti-NeuN (ab177487, Abcam), mouse anti-ubiquitin (sc-8017, Santa Cruz Biotechnology, Santa Cruz, CA), mouse anti-FLAG (#F1804, Millipore-Sigma), mouse anti-SQSTM1/p62 (ab56416, Abcam, Eugene) or rabbit anti-SQSTM1/p62 (CST-5411S, Cell Signaling Technology, Danvers, MA), mouse anti-UBQLN (#37-7700, Invitrogen, Carlsbad, CA), mouse anti-eIF2α (sc-133132, Santa Cruz Biotechnology), rabbit anti-phospho-eIF2α (#CST-3597L, Cell Signaling Technology), rabbit anti-p97/VCP (UMY475 home-made). Binding of primary antibodies was detected with the appropriate Alexa Fluor secondary antibodies (Invitrogen, Carlsbad, CA). Images were captured with a Hamamatsu digital C8484 camera attached to an inverted Leica DMIRB fluorescent microscope with either 10x, 20x, or 40x dry objectives using iVision software (BioVision Technologies, Exton, PA), or using a Nikon Eclipse Ti A1R confocal microscopy with a 60 × Apo oil objective and 405, 488 and 561 nm laser lines. Multi-wavelength fluorescent images of sections were merged using iVision software.

### Quantification of UBQLN2 inclusions

Brain and SC sections were stained with primary mouse anti-UBQLN2 antibody (#NBP2-25164, Novus Biologicals) followed by goat anti-mouse Alexa Fluor 488 nm secondary antibody. Digital images of the stained sections were captured with a 20× or 40× objective using the inverted Leica microscope. The number of UBQLN2-positive inclusions in identical areas of the CA1 and dentate gyrus regions of the hippocampus and of the anterior horn in the SC in different animals were estimated using iVision software to identify, quantify, and segregate the inclusions according to size. The data shown is the mean number of inclusions seen in three different animals for each genotype.

### Filter-retardation dot blot assay

Equal amounts of whole brain protein lysate (50 μg) were gently suctioned through a 0.2 μm cellulose acetate OE 66 membrane filter using a Biorad Dot blot apparatus connected to the house vacuum line as described previously [27, 32]. The filters were then probed for UBQLN2 protein essentially as described for immunoblotting.

### Brain neuronal counts by optical fractionator

Stereological principles based on the systematic random sampling method [34] were used to estimate the number of neurons in different regions of the hippocampus by analysis of coronal cut brain sections, as described before [14]. At least 10 brain sections (12 μm thick) were counted. The first section counted in the series was randomly selected from the first 20 sections containing the hippocampus. Thereafter, every 10th section was counted. NeuN-positive neurons contained in a predefined grid, 86.68 μm × 66.04 μm, in the CA1 and dentate gyrus regions of the hippocampus were counted as described previously [14]. The total population of neurons in the CA1 and dentate gyrus was estimated using the following formula: N = Q × (1/ssf) × (1/asf) × (1/tsf), where Q is the total number of neurons counted on the sampled sections, ssf is the section sample fraction, asf is area sampling fraction, and tsf is the thickness of the sampling fraction.

### Spinal MN counts

Quantification of MNs in the ventral horn of the SC was performed essentially as previously described [14]. Briefly, every 18th section (15 μm thick) of the lumbar enlargement section of the SC was stained with 1% cresyl violet in 0.25% glacial acetic acid, dehydrated with 70% and 100% ethanol, cleared with Histo-Clear (National Diagnostics, Atlanta, GA) and then coverslipped with DPX mounting medium (Electron Microscopy Sciences, Hatfield, PA). Images of the stained sections were digitally captured using a Qimaging Micropublisher 5.0 color camera with 4×, 10× and 20× objective attached on an inverted Olympus IX71 microscope. MNs in the entire ventral horn were counted manually using the optical dissected method [6]. MNs were only counted if they had cross-sectional area ≥ 250 μm^2^, had a multipolar shape with a distinct nucleolus, and had a darkly stained cytoplasm [6, 14]. The mean number of MNs in the ventral horn of a whole SC section for three animals is shown for each genotype.

### Muscle fiber diameter

The gastrocnemius muscle was dissected from 52 week-old animals and fixed and cyto-preserved before sectioning using the same procedure described for the brain [14]. Muscle sections were stained with hematoxylin and eosin (H&E) as described previously [14]. The average diameter of 40 muscle fibers for three independent animals for each of the four genotypes was determined using iVision software.

### Axon caliber

The number and cross sectional area of myelinated axons in the L4 ventral roots were quantified as described previously [14].

### RNA extraction and quantitative RT-PCR

Total RNA was extracted from the brain of 24-week-old mice using the Absolutely RNA Miniprep Kit (Agilent Technologies, Santa Clara, CA) according to the manufacturer’s instructions. The RNA was copied into cDNA using the high capacity cDNA Reverse Transcription kit with RNase inhibitor (ThermoFisher Scientific, Waltham, MA). Real Time-qPCR of three biological replicates for each sample was conducted with primers and probes, the properties of which are described below, with RotorGene 3000 software (QIAgen, Germantown, MD). Primers and probes were designed using IDT web software. Relative fold changes in expression were normalized to mouse β-actin and assessed using Pfaffl method and two-tailed t-test. The following primers were used:

mouse βAct: IDT PrimeTime qPCR Assay Mm. PT. 39a. 22214843. g (Forward: GATTACTGCTCTGGCTCCTAG; Reverse: GACTCATCGTACTCCTGCTTG; Probe:/5CY5/CTGGCCTCACTGTCCACCTTCC/IAbrQSp/); hUBQLN2: (Forward: CGATCCACTACCCAATCCATG; Reverse: ATAATTAGCGGCAGCAACGGTGT; Probe:/56-ROXN/TACCAGCACGACCACAAGCACT/3IAbRQSp/); hUBQLN1: (Forward: GGGTTTACAGACATTAGCAACG; Reverse: GCTGTGGGACTTGTGTTTTC; Probe:/5HEX/CACTGGAGG/ZEN/CTCTTCGGGAACTAA/3IABkFQ/).

### SDS-PAGE and immunoblotting

Tissues for protein blots were dissected from euthanized mice. Protein lysates were made from the lumbar region of the SC or one hemisphere of the brain by homogenizing the tissues in protein lysis buffer (50 mM Tris pH 6.8, 150 mM NaCl, 20 mM EDTA, 1 mM EGTA, 0.5% SDS, 0.5% NP40, 0.5% Sarkosyl, 10 mM sodium-orthovanodate, 2.5 mM sodium-fluoride and protease inhibitors). Protein concentration of the lysates were measured using bicinchoninic acid assay (BCA) (ThermoFisher Scientific). Equal amounts of the proteins were mixed with SDS sample buffer and heated for 5 min at 100 °C prior to separation by SDS-PAGE. The isolated proteins were transferred onto 0.45 μm PVDF membranes (Immobilon-P, Millipore, Billeria, MA) for 24 min at 2.5 V or 13 min at 5 V using the Invitrogen Power Blotter XL (ThermoFisher Scientific). The membranes were probed for different proteins with appropriate antibodies using the chemiluminescence method [31]. Primary antibodies against the following proteins were used; ubiquitin (sc-8017, Santa Cruz Biotechnology), FLAG (F1804-200UG, SIGMA), UBQLN2 (#NBP2-25164), SQSTM1/p62 (ab56416), all ubiquilins (#37-7700, Invitrogen), eIF2α (sc-133132, Santa Cruz Biotechnology), p-eIF2α (Cell Signaling), UBQLN2 (UMY75), TDP-43 (UMY112), p97/VCP (UMY475). Binding of the primary antibodies was detected with appropriate horse radish peroxidase-conjugated secondary antibodies. The chemiluminescence signals from the blots were captured with the Fluoro-Chem M imager (Protein Simple, San Jose, CA) and the intensity of different bands was quantified using either Image J or Alpha View software (Protein Simple).

### Statistical analysis

Student t-test and ANOVA tests were performed using MS Excel and GraphPad Prism 8.0 software (GraphPad Software Inc, San Diego, CA) for all statistical analyses. Error bars represent ± the standard deviation. Significant differences are denoted by an * for (P ≤ 0.05), by ** for (P ≤ 0.01) and by *** for (P ≤ 0.005).

## Results

### Generation of double transgenic mice coexpressing UBQLN1 and P497S mutant UBQLN2 proteins

We crossed congenic C57BL/6 Tg mouse lines expressing either human *UBQLN1* or the P497S ALS/FTD mutant *UBQLN2* cDNA with one another and obtained progeny with all four permutations of the two transgenes (Fig. 1b): Non-Tg, single transgenic UBQLN1, single Tg P497S UBQLN2 and double Tg UBQLN1:P497S UBQLN2 mice. The four genotypes were obtained in the expected Mendelian and sex frequencies. Because expression of both the UBQLN1 and P497S UBQLN2 transgenes are driven by the same Thy1.2 promoter we examined whether expression of the two transgenes differed in single and double Tg mice. Real-time quantitative PCR (RT qPCR) measurement of mRNA expression in 24-week-old animals revealed unaltered expression of the UBQLN1 and UBQLN2 transgenes in double Tg animals compared to single Tg animals (Fig. 1c). Their faithful expression in the double Tg animals enabled us to evaluate how UBQLN1 overexpression affects disease caused by the P497S mutant transgene.

**Fig. 2:**
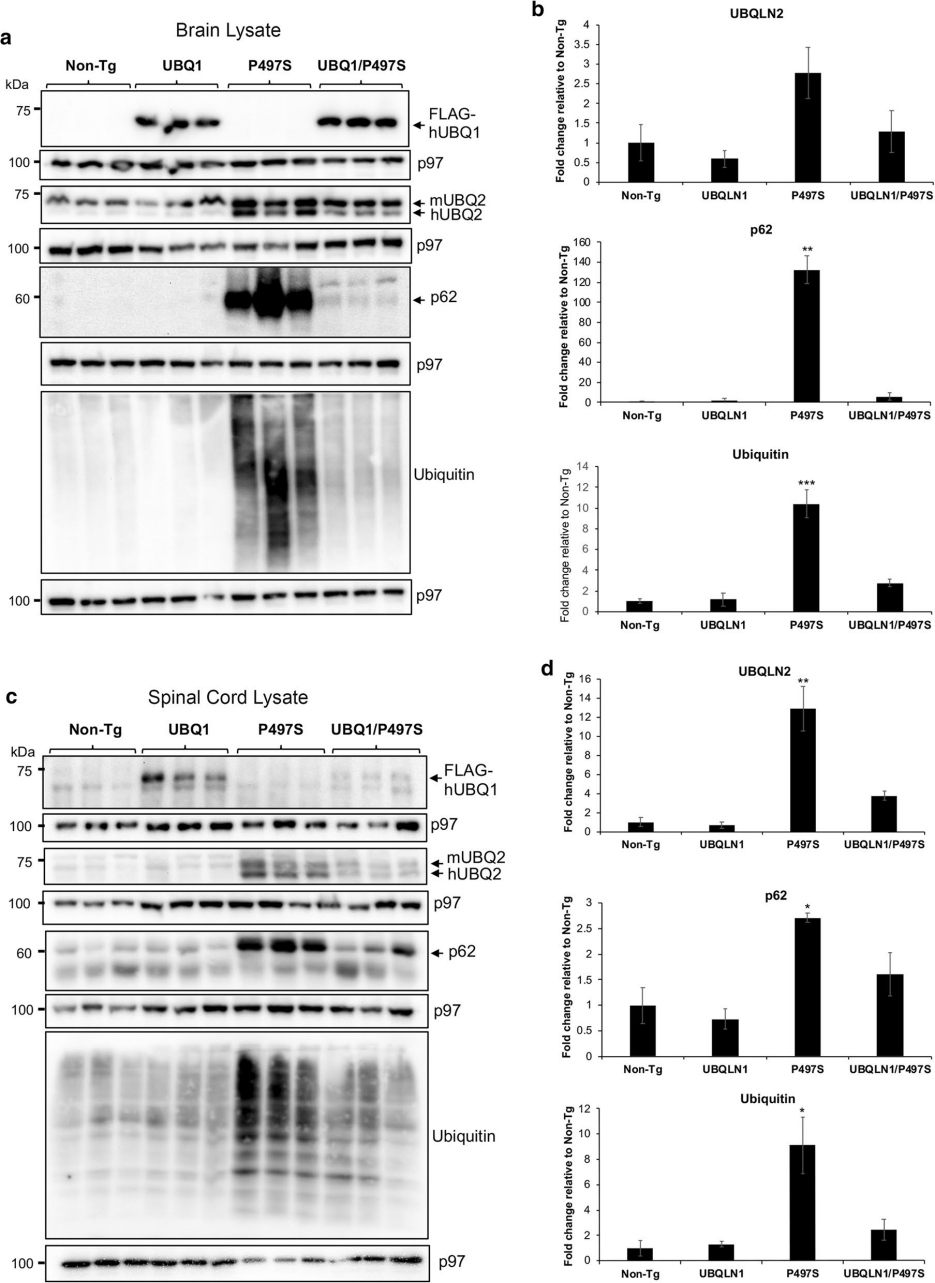
Immunoblots showing changes in proteins in the brain and SC of 52-week-old animals in each of the four genotypes. **a** Blots of equal amounts of protein lysate immunoblotted for the proteins shown and for p97/VCP as a loading control (2 males and 1 female for each genotype). **b** Quantification of the relative change in expression of the proteins from the blots shown in **a**. **P < 0.01, ***P < 0.001. **c**, **d** Blots were performed as in **a** and **b** except showing the results for protein lysates from the SC (2 males and 1 female for each genotype). *P < 0.05, **P < 0.01

Immunoblotting was used to analyze expression of the two Tg proteins in the four mouse genotypes. To ensure reproducibility of the findings, three different animals of the same age were analyzed for each genotype for this and most subsequent analyses. Expression of the transgenic human UBQLN1 protein was detected using an antibody specific for FLAG, which was used to tag the protein at its N-terminus [26], whereas expression of the P497S mutant human UBQLN2 protein was detected using a UBQLN2-specific antibody [14]. The Tg human UBQLN2 protein is distinguishable from the endogenous mouse UBQLN2 protein because of its smaller size [14]. Immunoblots of brain and SC lysates made from 52-week-old animals revealed expression of the appropriate Tg proteins in the correct genotypes (Fig. 2a, c). Interestingly, the total amount of endogenous and Tg UBQLN2 protein that accumulated in the brain and SC tissues in double Tg animals was lower than in single P497S mice (Fig. 2b, d). We reasoned that the reduction could have stemmed from reduced aggregation and/or accumulation of mutant UBQLN2 protein in the animals.

### Differential effects of UBQLN1 overexpression on behavior in double Tg male and female mice

**Fig. 3:**
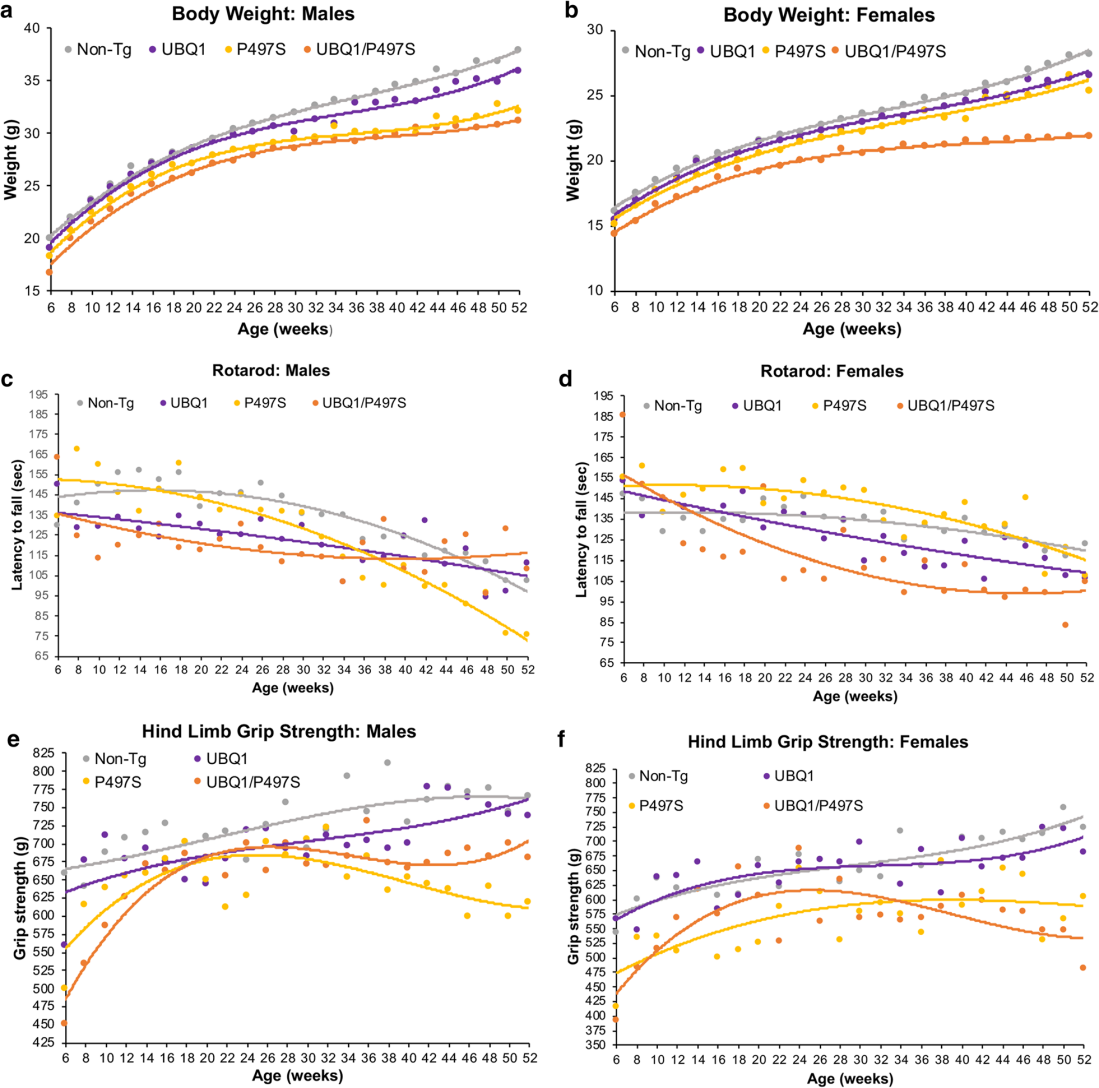
Effects of overexpression of UBQLN1 and P497S mutant UBQLN2 proteins on body weight and behavioral performance in male and female mice. **a**, **b** Graphs of the average whole body weight changes for the different genotypes conducted at 2 week intervals and the best-fit trend lines for both males (**a**) and females (**b**). Number of animals analyzed in each group: Non-Tg (n = 30 M, 28 F), UBQLN1 Tg (UBQ1) (n = 19 M, 19 F), P497S Tg (n = 20 M, 14 F), UBQ1/P497S (n = 8 M, 7 F). **c**, **d** Graphs of the rotarod analysis showing the average latency to fall for the different genotypes and best fit trend lines for both males (**c**) and females (**d**). Non-Tg (n = 13 M, 23 F), UBQ1 Tg (n = 9 M, 14 F), P497S Tg (n = 14 M, 17 F), UBQ1/P497S (n = 6 M, 6 F). **e**, **f** Graphs of the average hind limb grip strengths for the different genotypes and best fit trend lines for both males (**e**) and females (**f**). Non-Tg (n = 28 M, 27 F), UBQ1 Tg (n = 19 M, × 19), P497S Tg (n = 22 M, 16 F), UBQ1/P497S (n = 8 M, 7 F)

Mice of all four genotypes were tested by measuring their body weight, rotarod performance, and hind limb grip strength from 6 to 52 weeks of age (Fig. 3). Previous studies showed P497S mutant UBQLN2 mice can be discriminated from Non-Tg mice by age-dependent decline in these tests [14]. However, we were mindful that the P497S UBQLN2 line used for the current experiments had a more attenuated ALS-like phenotype compared to the original line. The attenuation occurred sometime during the repeated backcrossing of the original hybrid line with C57BL/6 mice. A similar attenuation in phenotype was also observed after backcrossing the G93A SOD1 mouse model for ALS into the C57BL6 genetic background [22]. Interestingly, the P506T UBQLN2 mouse model that we generated at the same time as the P497S model developed a worse phenotype after similar backcrossing and could not be bred for the current experiments.

All of the tests were conducted bi-weekly for cohorts of at least 5 animals for each genotype and gender (Fig. 3). The tests were stopped at 52 weeks because our preliminary studies indicated it was sufficient time to detect motor neuron loss in the P497S line. The tests revealed different effects of UBQLN1 overexpression that were gender-dependent, regardless of mutant UBQLN2 expression. Because of the complexity of the results, each will be described separately.

The body weight measurements indicated that single UBQLN1 Tg male and female mice were slightly lighter than Non-Tg mice, with a bigger difference seen at older age (~ 6% reduction in both males and females at 52 weeks of age) (Fig. 3a, b). A similar reduction in body weight was seen in Tg mice overexpressing UBQLN1 using the more ubiquitous CMV-actin promoter [17, 24]. By contrast, single P497S UBQLN2 Tg mice were even lighter than the single UBQLN1 Tg mice, with males having ~ 15% and females ~ 10% reduction in weight compared to the Non-Tg animals (Fig. 3a, b). Interestingly, the double Tg mice were the lightest of the four genotypes, with the males having ~ 18% reduction and females ~ 22% reduction in weight compared to Non-Tg animals.

Analysis of the rotarod data revealed a progressive age-dependent decline in latency to fall for all the genotypes, regardless of gender (Fig. 3c, d). However, major differences were found between the two sexes, and therefore their findings are described separately. To simplify the analysis, only the slopes of the rate of the change in performance are compared.

The changes in rotarod performance in the males (as well as for the females) were best fitted by polynomial trend lines. The duration of the latency to fall for single UBQLN1 Tg males was considerably shorter than Non-Tg animals, for all ages tested (Fig. 3c). However, the rate of decline in performance with age was slightly shallower for the UBQLN1 mice. By contrast, the rate of decline in performance for the single P497S Tg males was the steepest of all the genotypes, consistent with the idea that the mice develop MN disease (Fig. 3c). Interestingly, the rate of decline in rotarod performance in double Tg males was the slowest of all the groups, indicating UBQLN1 overexpression improved performance in mice expressing P497S mutant UBQLN2 protein (Fig. 3c).

The rotarod results for the female mice were notably different than for the males. The genotypes with the slowest and fastest rate of decline in performance were the Non-Tg and both P497S-containing genotypes, respectively (Fig. 3d). The declines were most noticeable after 30 weeks of age. The accelerated decline in the mutant P497S compared to the Non-Tg group is consistent with progressive development of motor neuron disease in the former. The smaller decline seen in Non-Tg animals may reflect gradual age-dependent decrease in body function. The single UBQLN1 Tg group had a steeper decline after the Non-Tg animals, but the difference was not very large. The double Tg group showed the most rapid rate of decline over time at early age (before 30 weeks), but after this period the decline became progressively shallow and appeared to slightly improve towards the end of the test period (Fig. 3d).

Analysis of the hind-limb grip strength were similar to the rotarod trends. Again, differences between the genders were apparent and the changes for both genders were again best fitted by polynomial trend lines (Fig. 3e, f). Analysis of the male results revealed single UBQLN1 Tg and Non-Tg mice had the highest grip strength of the four genotypes, both of which increased progressively at a similar rate with age (Fig. 3e). By contrast, the grip strength of single P497S Tg mice was generally lower than the UBQLN1 and Non-Tg animals. Their strength steadily increased till about 30 weeks of age, after which it rapidly declined, eventually decreasing by 25% compared to Non-Tg animals at 52 weeks of age (Fig. 3e). Double Tg mice were quite different. They had the weakest grip strength at early age (15% lower than Non-Tg animals), but this strength steadily increased, eventually surpassing that of single P497S Tg animals (Fig. 3e). At 52 weeks their grip strength was ~ 10% stronger than single P497S mice. These results when combined with the rotarod results strongly suggests that the strength and motor performance of double Tg male mice is superior than single Tg P497S animals.

Analysis of the grip strength for the female cohorts revealed a different pattern than the males. However, the data again showed single UBQLN1 Tg and Non-Tg groups had equivalent and the strongest grip strength of the four genotypes, both of which continuously increased with age (Fig. 3f). The single P497S Tg group had weaker grip strength than the single Tg UBQLN1 and Non-Tg groups for all ages tested. Their strength too increased progressively till about 30 weeks of age when it reached a plateau. Like the males, double Tg females had the weakest grip strength at early age, but their strength increased more rapidly with age than in any other group, till about 36 weeks of age, when it declined more rapidly than any other group (Fig. 3f).

Taken together the behavioral data indicated neuron-specific overexpression of UBQLN1 decreases body weight, irrespective of P497S transgene expression. Furthermore, the tests revealed a more pronounced male-specific benefit of UBQLN1 overexpression in alleviating rotarod and grip strength deficits typically seen in mice expressing the mutant P497S UBQLN2 transgene.

### Overexpression of UBQLN1 reduces accumulation of UBQLN2 inclusions and ubiquitinated proteins in the brain of P497S UBQLN2 Tg mice

**Fig. 4:**
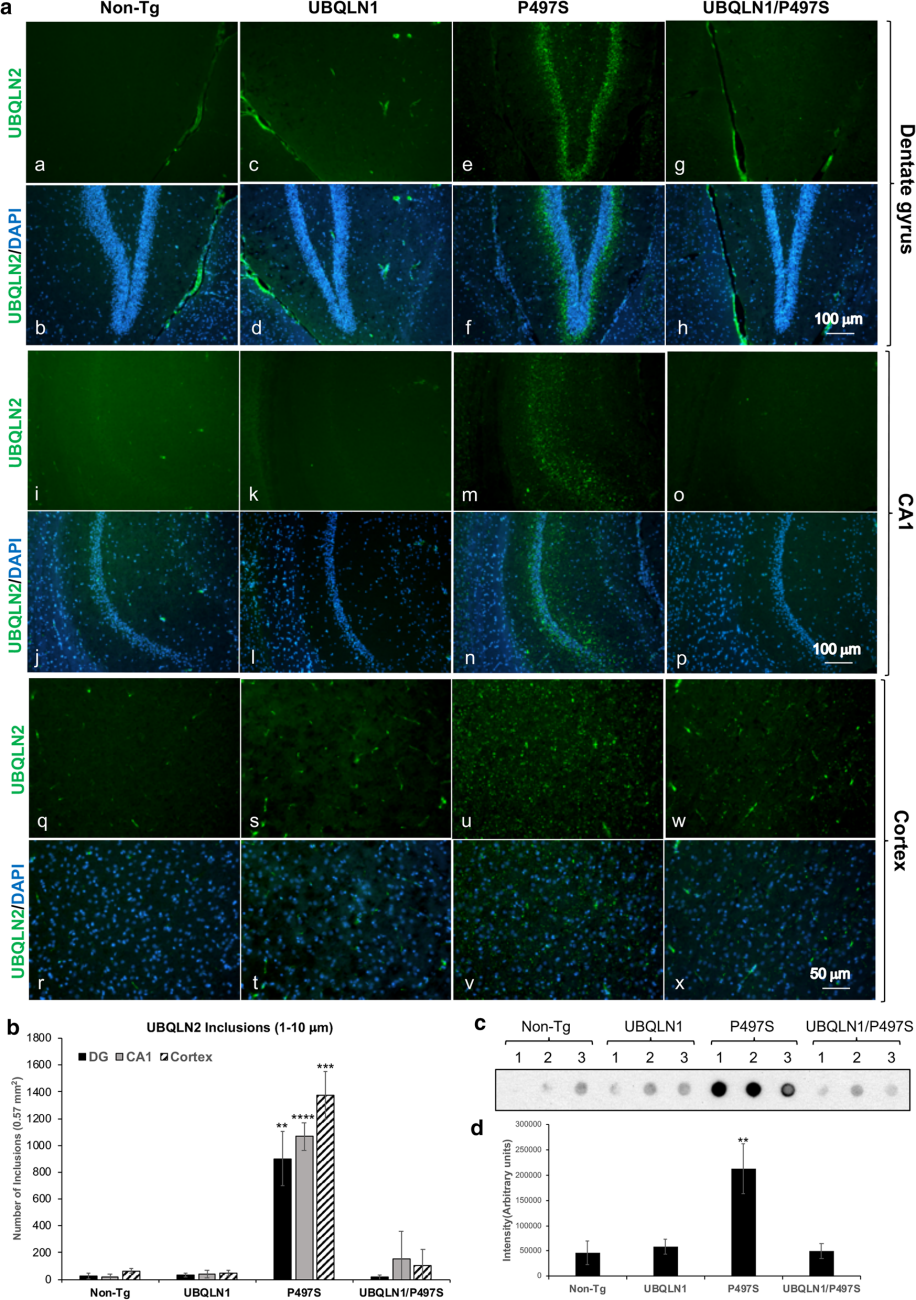
UBQLN1 overexpression reduces deposition of UBQLN2 inclusions compared to the brains of P497S mutant mice. **a** Staining of representative sagittal sections of the dentate gyrus (DG) (a–h), CA1 (i–p) and cortex (q–x) regions of the brain for UBQLN2 and DAPI in the four genotypes at 52 weeks of age. Scale bars shown. **b** Quantification of the number of UBQLN2 inclusions between 1 and 10 μm in size counted in identical size regions of the DG, CA1 and cortex regions (2 males and 1 female for each genotype). **P < 0.01, ***P < 0.001, ****P < 0.0001. **c** Filter-trap assay showing detection of UBQLN2 inclusions in 50 μg of protein brain lysate analyzed for the four genotypes for animals at 52 weeks of age. Samples analyzed were: Non-Tg, 2 male (M) and 1 female (F); UBQLN1 all F; P497S, 1 M, 2 F; UBQLN1/P497S all M. **d** Quantification of the intensity of UBQLN2 immunoreactivity for the filter shown in **c**. **P < 0.01

The four genotypes were examined for signatures of UBQLN2 pathology. Accordingly, brain sections of 52-week-old animals were stained for UBQLN2 inclusions [14]. As expected, single P497S Tg animals contained numerous UBQLN2-positive inclusions spread throughout the brain, with their characteristic high preponderance around the dentate gyrus of the hippocampus (Fig. 4a and Additional file 1: Fig. S1A) [14, 35]. These inclusions were considerably diminished in the double Tg animals. Quantification of the number of UBQLN2 inclusions separated by size (0.1–1 μm and 1–10 μm) in the dentate gyrus, CA1 and cortex regions of the brain revealed far fewer small and large-size inclusions in the three genotypes compared to the single P497S Tg animals (Fig. 4b and Additional file 1: Fig. S1). Intriguingly, the number of the large size inclusions were reduced in double Tg animals to almost the same levels as Non-Tg and UBQLN1 animals. However, some of the smaller size inclusions still persisted in the double Tg mice (Additional file 1: Fig S1B).

To confirm the reduction of UBQLN2 inclusions in mice overexpressing UBQLN1, we stained the brain sections for ubiquitin and p62/SQSTM1 (Additional file 1: Fig. S2A and B), as both proteins concentrate and colocalize with UBQLN2 inclusions in P497S animals [14, 35]. The results revealed, as expected, single P497S Tg animals contained numerous p62- and ubiquitin-positive foci, most of which colocalized with UBQLN2 staining (Additional file 1: Fig. S2A and B). By contrast, these foci were greatly reduced in the remaining genotypes, with the exception of the double Tg mice where smaller p62 inclusions were sometimes seen. Further analysis of whole brain lysates from the animals by a filter-retardation dot blot assay [27, 32] revealed double Tg mice also had reduced retention of UBQLN2 immunoreactivity on the filters compared to single P497S Tg animals (Fig. 4c). The immunoreactivity in the double Tg animals was comparable to that in Non-Tg and UBQLN1 mice, consistent with the idea they have fewer UBQLN2 aggregates (Fig. 4d).

We also probed whole brain lysates of each genotype for changes in UBQLN2, ubiquitin, and p62. Similar to the immunofluorescence findings, the blots for p62 and ubiquitin revealed dramatic reduction in accumulation and reactivity of both proteins in the double Tg mice compared to single P497S Tg animals (Fig. 2a, b). Taken together, these results indicate UBQLN1 overexpression reduces UBQLN2 inclusions in mice expressing the P497S UBQLN2 mutant transgene.

### Overexpression of UBQLN1 reduces neuronal loss in the brain of P497S UBQLN2 Tg mice

**Fig. 5:**
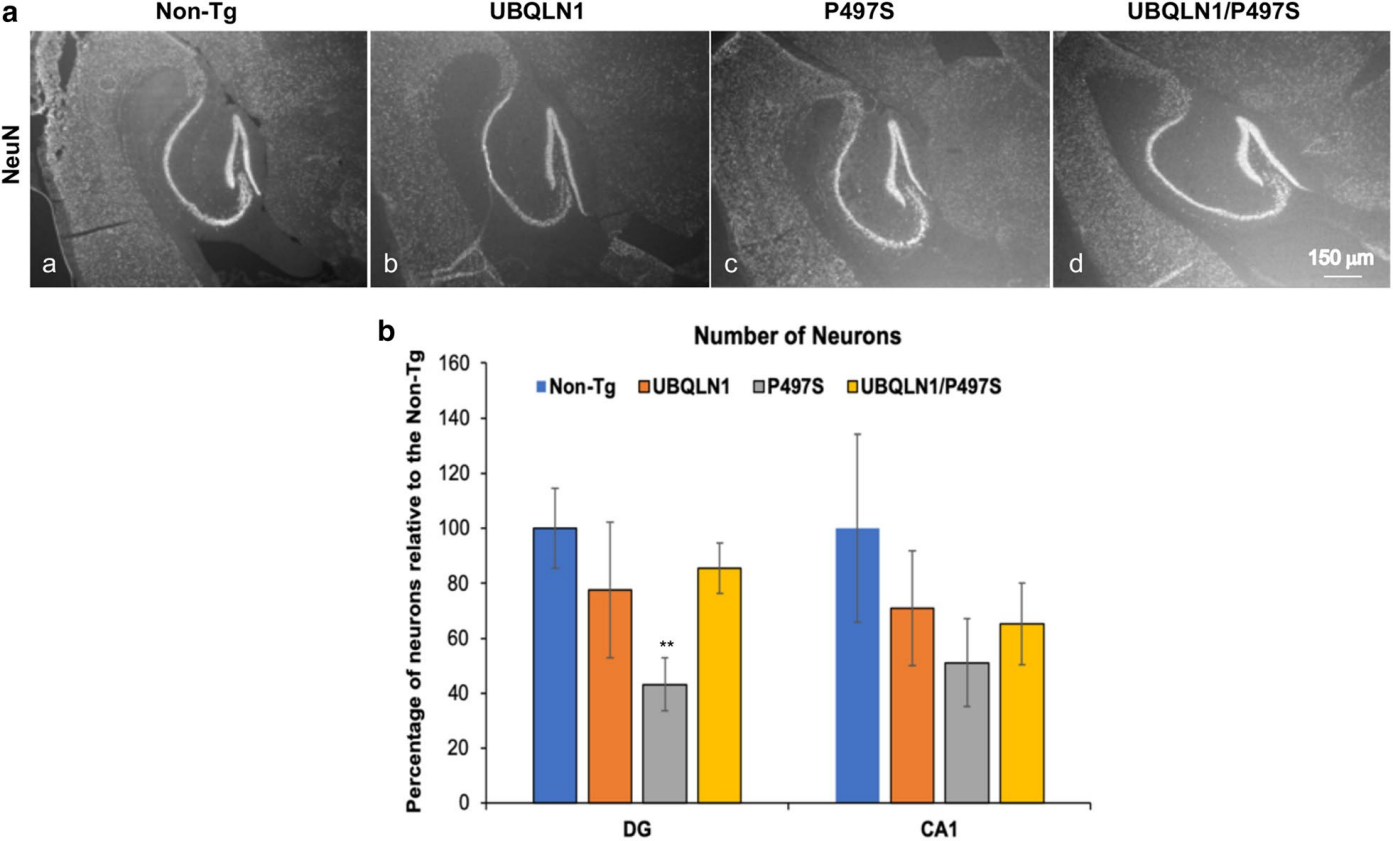
Neuronal loss in the hippocampus induced by overexpression of the P497S mutant UBQLN2 protein is partially rescued by UBQLN1 overexpression. **a** Representative examples of NeuN staining of the hippocampus regions for the different genotypes at 52 weeks of age. **b** Stereology neuron counts contained in similar size areas of the DG and CA1 regions of hippocampus for the different genotypes (2 males and 1 female for each genotype except the double Tg where 3 males were used). The reduction of neurons in the CA1 regions of P497S was close to significant (*P* = 0.07). **P < 0.01

A major neuropathologic consequence of overexpression of the mutant P497S transgene is loss of neurons in the hippocampus [14]. We therefore assessed whether UBQLN1 overexpression affects neuronal loss. Accordingly, we counted the number of NeuN-positive neurons in the CA1 and dentate gyrus regions in the four genotypes using stereological principles. The counts revealed, as expected, significant neuronal loss in the dentate gyrus in single P497S Tg animals at 52 weeks of age compared to Non-Tg and single UBQLN1 Tg animals (Fig. 5a, b). The quantifications also revealed a trend toward fewer neurons in the CA1 region of the P497S animals, but the reduction was just outside of significance (*P* = 0.07). We noted a slight trend in reduction of neurons in single UBQLN1 Tg mice in both the dentate gyrus and CA1 regions compared to Non-Tg mice, but the difference was not significant. More significantly, the quantifications revealed double Tg mice had reduced neuronal loss compared to single P497S Tg mice (Fig. 5b). Interestingly, the neuronal counts in the double Tg animals more closely resembled those found in single UBQLN1 Tg animals than in Non-Tg animals. Nevertheless, the results indicate UBQLN1 overexpression reduces neuronal loss in mice expressing the mutant P497S UBQLN2 protein.

### Overexpression of UBQLN1 reduces UBQLN2 inclusions and build-up of ubiquitinated proteins in the spinal cord of P497S Tg mice

**Fig. 6:**
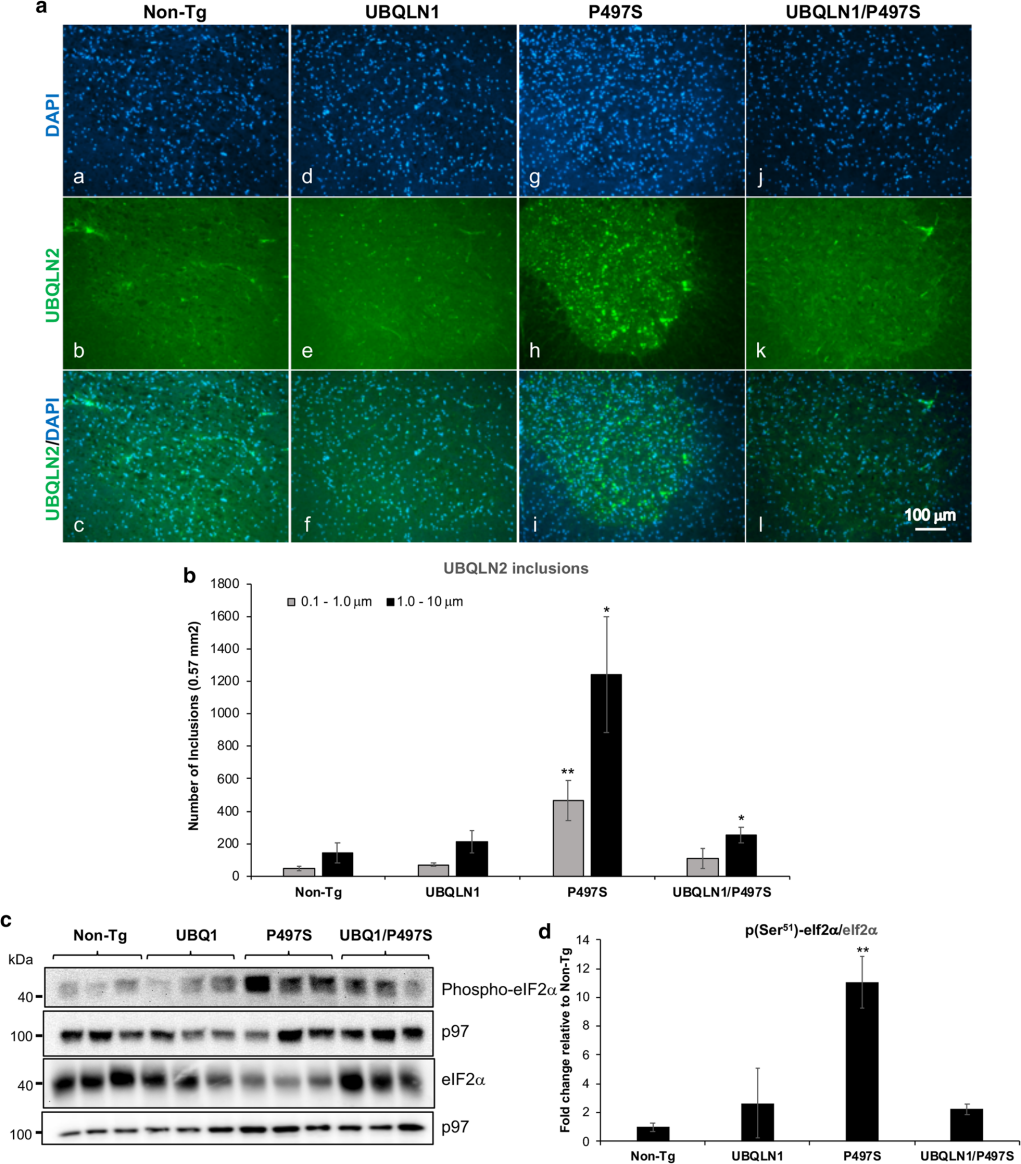
Overexpression of UBQLN1 reduces UBQLN2 inclusions and ER stress in the spinal cord. **a** Immunofluorescence staining of the sections in the ventral horn region of the spinal cord for UBQLN2 and DAPI and of their combined staining. **b** Quantification of the number of UBQLN2 inclusions based on size in the spinal cord of the four genotypes at 52 weeks of age (2 males and 1 female for each genotype). *P < 0.05, **P < 0.01. **c** Immunoblots of SC lysates for the different genotypes probed for the proteins shown. **d** Quantification of changes in phospho-eIF2α after normalizing for expression of total eIF2α protein (2 males and 1 female for each genotype). **P < 0.01

We next examined if the abrogation of UBQLN2 pathology found in the brain of double Tg mice extends to the spinal cord (SC), since expression of the mutant P497S transgene is associated with motor neuron disease [14]. Repetition of the blots using SC lysates revealed almost identical findings to those seen in the brain: a reduction in the double Tg animals of endogenous and transgenic UBQLN2 accumulation, decreased protein ubiquitination and reduction in p62 levels compared to single P497S animals (Fig. 2c, d). Immunostaining of the SC sections for UBQLN2 also revealed dramatic reduction in the number of UBQLN2 inclusions in the double Tg mice (Fig. 6a). Quantification of the reduction revealed double Tg mice still had significantly fewer small and large size 0.1–1 and 1–10 μm size inclusions compared to the P497S animals, despite having higher number of large size inclusions compared to Non-Tg animals (Fig. 6b).

Because UBQLN proteins function in ERAD, and ALS-FTD mutations interfere with the process [8, 37], we also probed the SC lysates for evidence of ER stress. Accordingly, we probed the samples for alteration in eIF2α phosphorylation (Ser^51^), an increase of which is reflective of increased ER stress [9, 28] (Fig. 6c). The blots revealed a strong increase in phospho-eIF2α(Ser^51^) levels in single P497S Tg animals compared to Non-Tg animals, which was considerably attenuated in the double Tg mice (Fig. 6c, d). Single UBQLN1 Tg mice had comparable phospho-eIF2α(Ser^51^) levels compared to the Non-Tg animals.

### Overexpression of UBQLN1 reduces MN loss and TDP-43 pathology in the SC

**Fig. 7:**
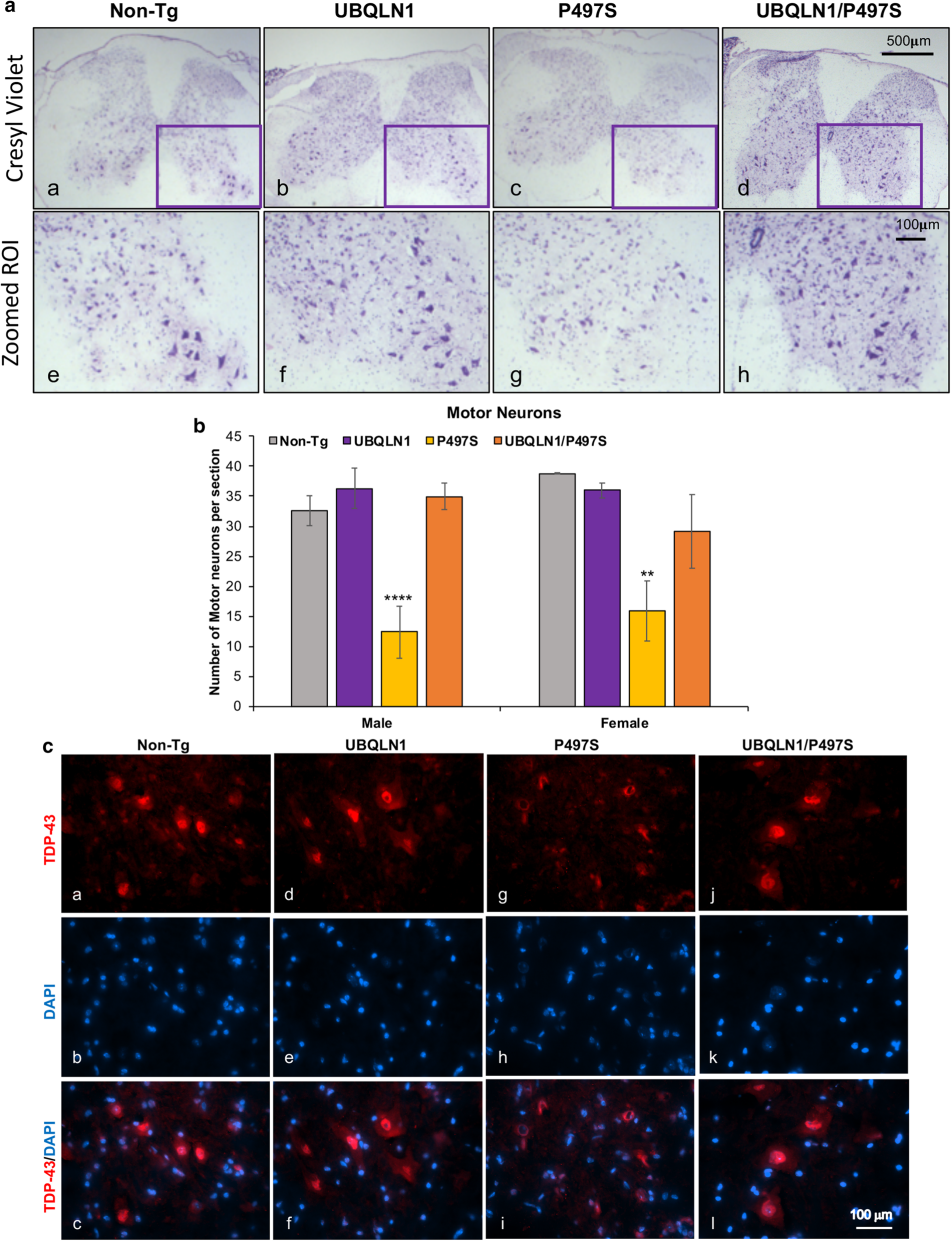
Overexpression of UBQLN1 reduces MN loss and TDP-43 pathology in mice expressing the P497S mutant UBQLN2 transgene. **a** Cresyl violet staining of the grey matter regions of the SC for the different genotypes (a–d). The magnified areas highlighted by the boxed regions are shown beneath each of them (e–h). **b** Quantification of the number of MN per section counted by stereology for the different genotypes (at least 3 animals for each genotype). **P < 0.01, ****P < 0.0001. **c** Immunofluorescence staining of TDP-43 and DAPI staining of the lumbar region of the SC for the different genotypes. Note TDP-43 staining is displaced from the nucleus to the cytoplasm in the P497S mutant Tg animal, but is restored to its typical location in the double Tg animals. The P497S section had more MN than typical and was used to better illustrate the TDP-43 pathology in the mice

Expression of the P497S mutant transgene is associated with age-dependent MN loss in the SC [14]. Accordingly, we quantified the number of MN in both male and female mice at 52 weeks of age for the four genotypes (Fig. 7a, b) [6, 14]. Changes in MN number was confirmed by ChAT staining of the sections (Additional file 1: Fig. S3). The quantification revealed little difference between UBQLN1 and Non-Tg animals. However, both male and female single P497S animals had approximately a 50% reduction in MNs (Fig. 7b). By contrast, MN number in double Tg male and female mice were very similar to their Non-Tg counterparts.

Further evidence supporting neuroprotection by UBQLN1 overexpression was found by examining TDP-43 pathology in spinal MN of the mice [14]. These examinations showed TDP-43 staining was restored to the nucleus in the double Tg mice compared to single P497S Tg mice, where many more MNs had displacement and accumulation of the staining in the cytoplasm (Fig. 7c). Single UBQLN1 Tg and Non-Tg animals showed no signs of TDP-43 pathology, as expected.

### Restoration of muscle weight and fiber size in double transgenic male UBQLN1/P497S Tg mice

**Fig. 8:**
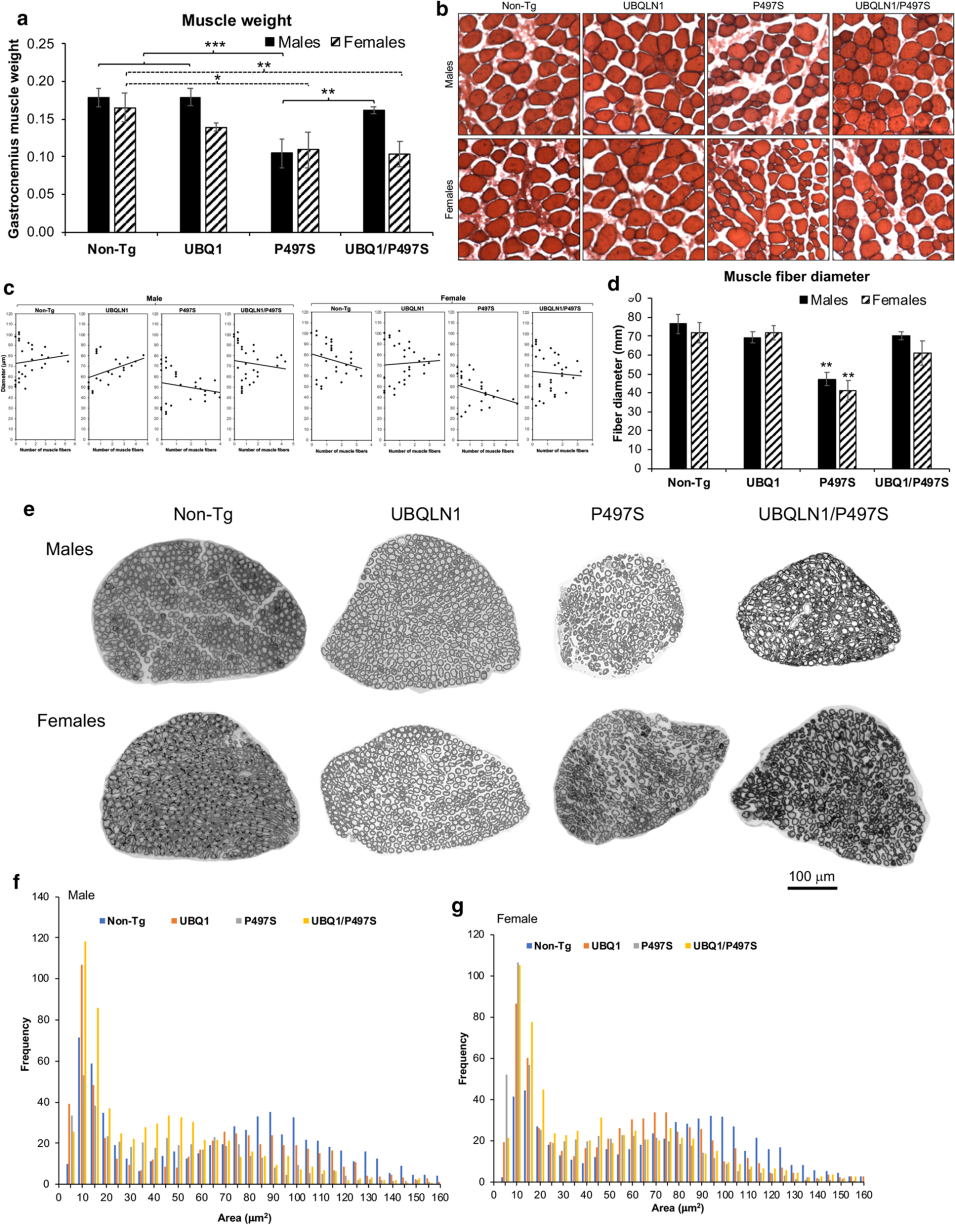
Muscle and axon properties of single and double UBQLN Tg and Non-Tg animals. **a** Gastrocnemius muscle weight (average for one limb for 3 different mice) at 52 weeks of age. **b** Transverse sections of the muscle stained with H&E. **c** Frequency distribution of muscle fiber size (n = 40) averaged for 3 different animals at 52 weeks of age. **d** Graph of the mean fiber size calculated from the data shown in **c**. **e** Changes in axonal number and caliber in L4 nerves of mice expressing the different UBQLN transgenes. **f** Representative transverse sections (1 μm) of the L4 ventral nerves isolated from male and female 52-week-old animals for the four genotypes. **b**, **c** Histograms of the mean frequency in distribution of myelinated axons grouped into 5 μm bins for the four genotypes in males (**b**) and females (**c**) (average of 3 animals for each genotype)

The weight of the gastrocnemius muscle and size of the muscle fibers was determined for the different animal cohorts at 52 weeks of age (Fig. 8). Measurement of muscle weight in male mice revealed an approximately 45% reduction in single P497S animals compared to Non-Tg animals, consistent with the expected weight loss from motor neuron disease in P497S animals (Fig. 8a). Muscle weight in single UBQLN1 mice was similar to Non-Tg animals. Importantly, its weight in double Tg male mice was similar to UBQLN1 and Non-Tg animals. The comparison of muscle fiber diameter in the male mice revealed a similar trend, with reduction of fiber size in single P497S animals and restoration to close to the normal size in double Tg animals (Fig. 8b–d).

Similar comparison of muscle weight and fiber size in the female cohorts revealed an increase in fiber size, but not muscle weight, in the double Tg mice (Fig. 8b–d). Like the males, muscle weight and fiber size were both reduced in the single P497S Tg female mice compared to UBQLN1 and Non-Tg animals. However, muscle weight was little different between double Tg and single P497S Tg female mice. Interestingly, however, muscle fiber diameter was increased in the double Tg female mice compared with P497S animals.

### UBQLN1 overexpression partially restores axonal caliber in male cohorts of double Tg mice

We next quantified the number and diameter of axons in the L4 nerve as an independent assessment of MN changes (Fig. 8a). Quantification of the total number of myelinated axons in transverse sections taken through the L4 nerve of 52-week-old animals revealed a near significant reduction in axon number in male P497S single Tg animals (*P* = 0.06) compared to Non-Tg animals (Additional file 1: Fig. S3A). Conversely, the numbers in double Tg and single UBQLN1 Tg male animals showed little difference compared to the Non-Tg animals (Additional file 1: Fig. S4A). Furthermore, the same comparison in female cohorts, revealed no difference across all four genotypes (Additional file 1: Fig. S4B).

The profile of the diameter of myelinated axons in both male and female mice showed the standard biphasic segregation of small (0–30 μm) and large (40–150 μm) caliber axons (Fig. 8b, c). However, we identified differences between the profiles of male and female mice. First, the peak of large caliber axons in male cohorts differed considerably across the four genotypes. In Non-Tg animals, it was centered at ~ 90–100 μm. By comparison, single UBQLN1 Tg mice centered at 70 μm, while double Tg mice centered at 40–50 μm. Single P497S Tg mice had the most flattened profile, suggesting the loss of large caliber axons (Fig. 8b). Comparison of the axon profile for the female groups revealed more similarity between the single UBQLN1 Tg and Non-Tg genotypes, although subtle differences were apparent (Fig. 8c). By contrast, both single and double Tg P497S animals had very similar profiles, with reduced number of large caliber axons and a pronounced increase in the cluster of small caliber axons (0–25 μm). The lack of alteration in the large caliber axons in double Tg animals suggests UBQLN1 overexpression did not rescue loss of large caliber axons in the double Tg female cohort. Taken together, examinations of the L4 axons provides additional support that overexpression of UBQLN1 can partially rescue MN loss in male mice expressing the P497S mutant transgene. However, the results also suggest that the benefit does not extend to females.

## Discussion

Here, we have shown that by crossing mouse lines expressing different UBQLN transgenes that overexpression of UBQLN1 alleviates neuropathology in the P497S UBQLN2 mouse model of ALS/FTD. The neuroprotective benefit was primarily evident by a reduction in pathologic features, but less so by changes in behavioral phenotypes in double Tg mice compared to single P497S Tg mice. We discuss the reasons why we place more weight on the pathologic rather than on the behavioral findings.

The reduction of pathologic features in double Tg mice was evident in all of the biochemical and cytological examinations we conducted of the animals, which showed reduced accumulation and/or build-up of UBQLN2 protein inclusions, ubiquitinated proteins, and p62 and ER stress markers, as well as restoration of muscle fiber size, TDP-43 nuclear localization in spinal motor neurons and of axonal number in L4 nerves, compared to single P497S Tg mice. Improvements were found across the brain and SC tissues of the animals, suggesting that the neuroprotective benefit of UBQLN1 overexpression extends throughout the central nervous system (CNS). This widespread neuroprotective effect is consistent with the Thy1.2 promoter-driven expression pattern of the UBQLN1 and 2 transgenes used in the study.

One simple explanation that we discounted is that the reduction in pathology in double Tg mice stems from reduced expression of the mutant P497S transgene compared to single P497S Tg mice. Expression of both the UBQLN1 and P497S transgenes were placed under control of the same Thy1.2 promoter, and therefore any competition of the promoters for transcription factors could have resulted in reduction in expression of one or both transgenes. However, RT qPCR measurement of mRNA expression revealed that neither expression of the mutant P497S UBQLN2 nor the WT UBQLN1 transgenes were altered in double Tg animals compared to their expression level in single Tg animals. Therefore, the reduced pathology in double Tg mice is more likely a consequence of increased UBQLN1 expression rather than reduced expression of the mutant P497S transgene.

Despite the sharp reduction in pathologic features, the double Tg mice did not display marked improvement in behavioral phenotypes when compared to the single P497S Tg animals. However, closer inspection of the trend lines for the behavioral phenotypes indicated a gender-specific benefit that was mainly noticeable in males. For example, male double Tg animals had better rotarod performance and stronger grip strength than single P497S Tg animals at older ages (40–52 weeks of age), corresponding to when MN disease symptoms should have manifested. By comparison, female double Tg mice also exhibited slight improvement in rotarod performance, but not in grip strength. However, we caution that the differences we found are based on analysis of small group sizes of animals and will require confirmation using larger group size of animals and/or a longer testing periods.

One confounding factor, whose contribution we cannot assess in affecting the behavioral results, is the influence that body weight changes have on them. As noted, the body weights of animals for the different genotypes differed greatly. For example, Tg mice overexpressing either WT UBQLN1 or mutant UBQLN2 proteins had reduced body weight compared to Non-Tg animals, with double Tg mice showing the greatest reduction of the four genotypes. It is unclear from our results whether these changes reflect beneficial or detrimental effects of UBQLN overexpression. There are reasons to believe that both could be involved.

For example, a previous study reported that global overexpression of UBQLN1 is associated with a reduction in mouse body weight [24]. The slight reduction in body weight seen in our male and female single Tg UBQLN1 animals is consistent with this prior observation. Besides the alteration in body weight, we found little evidence that overexpression of UBQLN1 by itself was toxic. For example, all of the biochemical and cytological characterizations of the single UBQLN1 Tg animals showed no indication of increased pathology. The only observed difference was a noticeable shift in large caliber axons to slightly smaller axons in L4 nerves. However, the total number of axons in the nerves was unaltered suggesting little neuronal loss. This was further supported by lack of differences in MN number in the SC and neurons in the dentate gyrus and CA1 regions of the hippocampus between UBQLN1 single Tg and Non-Tg animals.

Single P497S Tg animals also exhibited body weight loss compared to Non-Tg animals, with males displaying greater loss than females. One simple explanation for the gender difference is expression of the P497S transgene differs in male and female mice. However, RT-qPCR measurement of mRNA expression in 24-week-old animals revealed similar level of expression of the P497S transgene in the two sexes (Fig. 1d), eliminating this possibility. Another possibility is that expression of the P497S mutant transgene is more penetrant in males than in females because of differences in the number and expression of the UBQLN2 alleles in the two sexes. Support for such a hypothesis is seen in human carriers of UBQLN2 mutations. For example, male carriers of certain *UBQLN2* mutations tend to develop disease much earlier than their female siblings (reviewed by [10]). Because the *UBQLN2* gene, both in humans and mice, resides on the X chromosome, an obvious explanation for the delay in females would be the presence of two X chromosomes. Accordingly, males will carry only the mutant *UBQLN2* allele, whereas females can carry both a mutant and a WT *UBQLN2* allele. It is possible, although unproven, that the contribution of the normal WT *UBQLN2* allele “dilutes” the effect of the mutant *UBQLN2* allele in females, thereby slowing disease. Obviously, X-chromosome inactivation could further impinge on this penetrance. If this reasoning is correct, it may provide an explanation for why male single P497S Tg mice have accelerated weight loss compared to females, because the former only has one while the latter has two copies of the mouse *UBQLN2* gene to dilute out the human mutant *UBQLN2* transgene.

A third possibility is the weight loss is more related to the total amount of UBQLN protein expressed in the animals regardless of isotype, or whether it is a WT or a mutant protein. Support for this possibility are the effects produced by overexpression of WT UBQLN1 found in this study and the reduction in body weight seen in animals overexpressing WT UBQLN2 [14]. This could also explain why both male and female double Tg mice had the greatest reduction in body weight compared to the other three genotypes, despite clear evidence for reduced neuropathology in males. On the other hand, the poorer performance of the double Tg females in the behavioral tests could suggest too much UBQLN expression is toxic thus inducing disease and the corresponding greater weight loss. If true, the toxicity could originate from having too much UBQLN1, too much UBQLN2, or the resulting effects of having too much of both proteins. We do not believe that overexpression of UBQLN1, per se, is toxic as several studies that have shown high overexpression of the protein in either neurons or throughout the whole body is not only non-toxic, but is in fact beneficial in alleviating disease pathology in mouse models of Huntington’s disease, Alzheimer’s disease and an ischemic stroke model of injury [1, 17, 26]. Furthermore, as already mentioned, we did not observe pathologic features in female mice overexpressing UBQLN1. On the other hand, studies have shown that high overexpression of UBQLN2 can be toxic in animals [12]. It is therefore conceivable that in female mice the threshold of UBQLN protein overexpressed was exceeded (from overexpression of the two different transgenes and the two endogenous mouse *UBQLN2* genes).

The ambiguity in interpretation of the behavioral results in female mice contrasts with most of the pathologic findings showing overexpression of UBQLN1 can alleviate disease caused by the P497S mutant UBQLN2 protein. Although the mechanism by which overexpression of UBQLN1 leads to the neuroprotection is not known, we speculate on likely possibilities based on the pathologic findings and known properties of UBQLN1 protein. A key factor that we believe is tied to the neuroprotection observed upon UBQLN1 overexpression in P497S expressing animals is the reduction in both mutant UBQLN2 protein accumulation and the burden of inclusions in the double Tg animals. Support for this conclusion comes from two key observations. First, the reduction of UBQLN2 inclusions was directly related to less neuronal loss in the animals. Second, longitudinal studies of neuronal survival had found formation of UBQLN2 aggregates is tightly linked to cell death [29]. The mechanism by which UBQLN1 overexpression reduces UBQLN2 levels and aggregates is not known. We speculate on several likely, but not mutually exclusive, possibilities. One, UBQLN1 could compensate for loss of normal UBQLN function induced by expression of the dominant P497S mutant UBQLN2 protein. There is strong likelihood of this possibility as UBQLN1 and 2 have both been shown to function in autophagy and proteasome degradation pathways [16, 21, 25, 35]. In this scenario, any aggregation of UBQLN2 aggregates may themselves be cleared through the autophagy and/or proteasome pathways. Two, UBQLN1 could directly target the misfolded UBQLN2 protein for degradation. Three, UBQLN1 could dimerize or oligomerize with mutant UBQLN2 proteins, reducing its toxicity.

A good biochemical indicator that the health of double Tg animals was better than single P497S Tg animals was the improvement seen in proteostasis, as evidenced by a reduction of ubiquitin protein buildup, p62 accumulation, and ER stress. These improvements are consistent with the role UBQLN1 proteins are known to play in maintaining protein quality control, by facilitating proteasomal degradation, autophagy and ERAD [16, 21, 25, 32, 33]. This improvement is also similar to the benefits found upon overexpression of UBQLN1 in other mouse models of neurodegeneration [1, 17, 18, 26].

Finally, our findings showing that UBQLN1 overexpression alleviates pathogenicity caused by UBQLN2 mutations, at least in males, may have considerable implications for therapeutic intervention. It suggests that devising methods to increase UBQLN1 expression could delay and possibly prevent disease caused by UBQLN2 mutations. However, we caution that due to the dramatic reduction of body weight seen in female mice from UBQLN1 overexpression, any contemplation of such an approach will have to be carefully controlled to ensure the optimal amount of UBQLN1 is overexpressed in order to prevent any potential detrimental effects. Clearly, further studies are needed to validate the usefulness of such an approach for ALS/FTD therapy.

## Supplementary information

**Additional file 1.** Supplemental information containing additional data for Overexpression of UBQLN1 reduces neuropathology in the P497S UBQLN2 mouse model of ALS/FTD.

## Data Availability

All the data used for the study is contained in the article or files in the supplemental information. All other datasets used in the study are available from the corresponding author.
