# Supplementary material for: Overexpression of UBQLN1 reduces neuropathology in the P497S UBQLN2 mouse model of ALS/FTD
Source: Acta Neuropathol Commun. 2020 Oct 7;8:164. doi: 10.1186/s40478-020-01039-9 (PMC7539388; doi:10.1186/s40478-020-01039-9)
Supplement: Supplementary file 1 — Additional file 1. Supplemental information containing additional data for Overexpression of UBQLN1 reduces neuropathology in the P497S UBQLN2 mouse model of ALS/FTD. [file 40478_2020_1039_MOESM1_ESM.pdf]

Supplemental Data

Tables

Table 1: Behavioral data showing the mean and P values for the different genotypes for the data shown in Fig 3.

Table 2: Means of the frequency and P value comparison of the L4 axon sizes in the different genotypes shown in Fig 8F and G.

Figures

Fig S1. UBQLN1 overexpression reduces deposition of UBQLN2 inclusions in the brain of P497S mutant mice. (A) Higher magnification images of the dentate gyrus regions of the brain in the different mouse genotypes to better illustrate the deposition and staining of UBQLN2 in the tissues. Scale bar shown. (B) Quantification of the number of UBQLN2 inclusions between 0.0 to 1 μm in size counted in identical size regions of the DG, CA1 and cortex regions (2 males and 1 female for each genotype). ***$P < 0.001$, ****$P < 0.0001$.

Fig S2. UBQLN1 overexpression reduces accumulation of p62 and ubiquitin-positive inclusions in mice expressing the P497S transgene. (A) Confocal images of the dentate gyrus regions (1X and 5X zoomed) of the hippocampus stained for p62, UBQLN2 and DAPI for the different genotypes. (B) Same as A except stained for ubiquitin instead of p62. Scale bars shown.

Fig S3. Detection of MN cells in the SC sections of the different mouse genotypes. SC sections were stained with an anti-ChAT antibody to identify MN in the different mouse genotypes. The MNs identified by ChAT staining directly correlated with the number quantified by cresyl violet staining.

Fig S4. Quantification of the number of myelinated axons in the L4 ventral nerve for male and female mice for the four genotypes. (A) Quantification of all myelinated axons in the L4 nerve in the male mice at 52 weeks of age. (B) Same as A, except for females.

Table 1: Behavioral Data Showing the Mean and P Values

Weight Females		Means Non-Tg	Means UBQ1	Means P497S	Means UBQ1/P497S	P values Non-Tg vs UBQ1	P values Non-Tg vs P497S	P values Non-Tg vs UB1/P497S	P values UBQ1 vs P497S	P values UBQ1 vs UB1/P497S	P values P497S vs UB1/P497S
	6 wks	16.06	15.43	14.87	14.3	0.140137	0.007363	0.014029	0.132891	0.060803	0.311053
	8 wks	17.42	16.94	16.36	15.32	0.303487	0.015902	0.002318	0.220527	0.027702	0.025824
	10 wks	18.4	17.71	17.58	16.56	0.111564	0.053347	0.001749	0.764553	0.05461	0.0287
	12 wks	19.3	18.8	18.42	17.15	0.260856	0.079615	0.001948	0.387628	0.004959	0.031523
	14 wks	20.1	19.84	18.71	17.7	0.597506	0.006653	0.001025	0.022694	0.002496	0.049378
	16 wks	20.48	20.08	19.5	18.64	0.378303	0.050171	0.002251	0.317635	0.035107	0.21014
	18 wks	20.75	20.61	19.86	19.26	0.772766	0.051839	0.016322	0.19218	0.087244	0.291853
	20 wks	21.46	21.36	20.44	19.13	0.867621	0.054413	0.0028	0.137774	0.013916	0.046249
	22 wks	21.87	21.46	20.59	19.48	0.474263	0.023186	0.001486	0.138826	0.009567	0.059168
	24 wks	22.17	21.64	21.18	19.93	0.385745	0.085881	0.00403	0.491417	0.056908	0.085917
	26 wks	22.68	22.21	21.53	19.91	0.468221	0.08518	0.002963	0.307884	0.009223	0.036296
	28 wks	23.12	22.32	21.92	20.8	0.202126	0.067789	0.012475	0.543319	0.092489	0.132726
	30 wks	23.52	22.91	21.95	20.46	0.384275	0.031992	0.003316	0.159236	0.007306	0.053312
	32 wks	23.76	23.29	22.44	20.69	0.499289	0.044889	0.001837	0.221047	0.011481	0.013945
	34 wks	24.23	23.32	22.88	21.12	0.365867	0.152686	0.022976	0.65497	0.09442	0.122329
	36 wks	24.76	23.8	22.96	21.05	0.263572	0.045684	0.005894	0.322724	0.018568	0.063665
	38 wks	24.8	24.13	23.04	21.12	0.435193	0.049761	0.002442	0.284615	0.027523	0.046531
	40 wks	25.07	24.53	22.71	21.45	0.523804	0.029764	0.004293	0.049563	0.00434	0.120989
	42 wks	25.78	25.21	24.53	21.5	0.547588	0.245562	0.001629	0.602322	0.021461	0.022439
	44 wks	25.9	24.78	24.79	21.53	0.355521	0.417488	0.011809	0.993867	0.036396	0.05462
	46 wks	26.9	26.13	24.66	21.65	0.532056	0.103751	0.003566	0.29088	0.008854	0.068071
	48 wks	27.33	26.09	25.31	21.72	0.264691	0.15233	0.002163	0.556558	0.00661	0.052721
	50 wks	27.97	26.22	26.22	21.75	0.124294	0.203597	0.000435	0.998069	0.007281	0.010154
	52 wks	28.14	26.5	24.93	21.78	0.166191	0.02781	0.000695	0.270751	0.006928	0.072438

Weight Males		Means Non-Tg	Means UBQ1	Means P497S	Means UBQ1/P497S	P values Non-Tg vs UBQ1	P values Non-Tg vs P497S	P values Non-Tg vs UB1/P497S	P values UBQ1 vs P497S	P values UBQ1 vs UB1/P497S	P values P497S vs UB1/P497S
	6 wks	19.86	18.62	18.26	16.64	0.028217	0.001466	0.000007	0.591658	0.036835	0.046615
	8 wks	21.94	20.96	20.77	19.92	0.128235	0.065432	0.008906	0.807525	0.26429	0.344285
	10 wks	23.61	23.01	22.41	21.45	0.354408	0.087613	0.020016	0.3898	0.063135	0.319343
	12 wks	25.08	24.27	23.59	22.73	0.253814	0.070394	0.021599	0.345764	0.055515	0.402924
	14 wks	26.7	25.25	24.81	24.18	0.083131	0.027608	0.027756	0.549977	0.227102	0.522356
	16 wks	27.1	26.41	26.02	25.02	0.384989	0.177964	0.055145	0.603418	0.124162	0.297335
	18 wks	28.07	27.26	26.86	25.52	0.317496	0.158273	0.024039	0.605366	0.042923	0.195558
	20 wks	28.46	27.79	27.06	26.15	0.473171	0.113901	0.073994	0.402695	0.178854	0.445794
	22 wks	29.32	28.44	27.76	27.02	0.346632	0.098	0.085762	0.388273	0.172627	0.506185
	24 wks	30.29	29.11	28.36	27.23	0.21537	0.033012	0.019241	0.36017	0.100516	0.303476
	26 wks	30.87	30.06	28.38	27.8	0.453342	0.018925	0.055598	0.06597	0.09929	0.632847
	28 wks	31.35	30.61	29.03	28.48	0.46479	0.026037	0.066294	0.074632	0.111381	0.678877
	30 wks	31.94	29.99	29.41	28.5	0.075297	0.005106	0.015262	0.562203	0.353343	0.447047
	32 wks	32.5	31.22	29.5	29.3	0.214819	0.003046	0.041972	0.05013	0.163193	0.868273
	34 wks	33.07	30.8	30.57	29.4	0.085675	0.056601	0.034	0.857975	0.401611	0.40513
	36 wks	33.15	32.82	30.02	29.09	0.796776	0.008764	0.021094	0.016312	0.026018	0.491561
	38 wks	33.85	32.76	30.1	29.53	0.381825	0.001253	0.009679	0.03101	0.062646	0.681672
	40 wks	34.51	33.04	29.86	29.84	0.272016	0.000511	0.009443	0.01919	0.080801	0.987672
	42 wks	34.82	32.97	29.96	30.39	0.212919	0.000259	0.017528	0.036932	0.214036	0.769285
	44 wks	35.92	34	31.51	30.5	0.198154	0.005431	0.009001	0.126551	0.082839	0.626284
	46 wks	35.53	34.72	31.28	30.55	0.596918	0.007916	0.01443	0.038549	0.046496	0.719955
	48 wks	36.77	35.09	31.51	30.38	0.284313	0.000772	0.002364	0.032722	0.036858	0.504615
	50 wks	36.76	35.62	32.61	30.74	0.477334	0.010011	0.003012	0.109717	0.038594	0.326233
	52 wks	37.8	35.85	31.96	31.05	0.261303	0.001633	0.002713	0.071361	0.068805	0.663055

Rotarod Females		Means Non-Tg	Means UBQ1	Means P497S	Means UBQ1/P497S	P values Non-Tg vs UBQ1	P values Non-Tg vs P497S	P values Non-Tg vs UB1/P497S	P values UBQ1 vs P497S	P values UBQ1 vs UB1/P497S	P values P497S vs UB1/P497S
	6 wks	146.8	153.4	162	185	0.5301	0.112726	0.012545	0.453824	0.07767	0.151848
	8 wks	144.6	135.8	161.4	151.2	0.382294	0.197245	0.706993	0.121671	0.462621	0.699991
	10 wks	128.7	144.3	135.8	144.7	0.140925	0.576742	0.239558	0.600696	0.981522	0.695981
	12 wks	135.1	140.1	146.1	122.7	0.614835	0.330042	0.345429	0.62571	0.126827	0.166255
	14 wks	128.6	138.6	143.6	119.6	0.276864	0.272591	0.520597	0.744844	0.117246	0.335806
	16 wks	134	140.6	154.4	115.8	0.491475	0.07307	0.20062	0.269313	0.078977	0.04568
	18 wks	133.5	148.2	152.9	118.2	0.094849	0.062547	0.248069	0.669014	0.01704	0.056428
	20 wks	144.3	130.3	139.3	150.2	0.242605	0.680598	0.741012	0.509853	0.283697	0.6037
	22 wks	140.3	137.8	136.9	105.3	0.806037	0.784614	0.027848	0.949567	0.045775	0.1561
	24 wks	145.5	136.8	151.4	109.4	0.394747	0.6227	0.031021	0.226895	0.021647	0.036585
	26 wks	135.4	124.8	140.3	105.2	0.354365	0.598641	0.099651	0.086516	0.288593	0.014614
	28 wks	133.7	134.6	144.1	128.8	0.935412	0.406053	0.766369	0.487314	0.692107	0.468929
	30 wks	135.5	114.2	137.3	110.6	0.06271	0.875361	0.178925	0.046483	0.806972	0.15791
	32 wks	137.6	125.9	132.7	114.6	0.306136	0.684076	0.204743	0.615967	0.529412	0.399941
	34 wks	124.1	117.5	123.3	99	0.541425	0.943334	0.20046	0.637703	0.324866	0.282871
	36 wks	131.6	111	129.1	114	0.046653	0.864173	0.293183	0.299881	0.848218	0.619248
	38 wks	128.2	112.1	128.9	99.25	0.098607	0.951978	0.100512	0.208153	0.472016	0.22993
	40 wks	134.9	123.6	141.6	112.3	0.25529	0.568199	0.146631	0.250104	0.575861	0.247368
	42 wks	127.6	105.6	126.8	100	0.013531	0.932816	0.12211	0.072917	0.783049	0.27445
	44 wks	130.9	125.5	136.6	96.25	0.596069	0.624487	0.042799	0.325039	0.029649	0.036566
	46 wks	124.3	121.2	138.3	100.3	0.788032	0.313793	0.244557	0.236428	0.270261	0.159853
	48 wks	118.9	115.5	110.5	98.75	0.750158	0.456382	0.224775	0.709644	0.384739	0.509091
	50 wks	116.8	106.9	118.9	83	0.359364	0.849815	0.059811	0.288765	0.177399	0.037888
	52 wks	122.8	106.2	108	104	0.07925	0.244346	0.148485	0.912944	0.906435	0.881471

Rotarod Males		Means Non-Tg	Means UBQ1	Means P497S	Means UBQ1/P497S	P values Non-Tg vs UBQ1	P values Non-Tg vs P497S	P values Non-Tg vs UB1/P497S	P values UBQ1 vs P497S	P values UBQ1 vs UB1/P497S	P values P497S vs UB1/P497S
	6 wks	129.6	149.5	134	163.3	0.055674	0.698694	0.003487	0.281617	0.29072	0.053624
	8 wks	140.4	128.3	167.4	124.3	0.398738	0.066229	0.322796	0.014506	0.796605	0.016105
	10 wks	149.8	128.8	159.7	112.9	0.040631	0.415027	0.002397	0.023757	0.097464	0.002757
	12 wks	155.3	133.2	145.5	119.3	0.106372	0.489981	0.015938	0.386753	0.283624	0.087134
	14 wks	156.8	127.8	136.4	124.3	0.026334	0.06938	0.027308	0.477905	0.818295	0.375153
	16 wks	152.2	123.4	147.4	130	0.008925	0.651028	0.074764	0.033379	0.563064	0.1706
	18 wks	155.5	133.8	160.3	118	0.075657	0.73621	0.005597	0.097372	0.14223	0.01574
	20 wks	138.4	129.7	143.4	117.2	0.473519	0.741456	0.18406	0.429261	0.372081	0.200695
	22 wks	145.1	124.4	136.6	122.3	0.096179	0.552314	0.141049	0.421502	0.881124	0.422088
	24 wks	145.5	124.7	144.6	129.6	0.172477	0.944246	0.23799	0.250713	0.777402	0.335337
	26 wks	150.1	132.3	137.1	118.2	0.246513	0.242749	0.058572	0.709178	0.462142	0.176923
	28 wks	143.9	122.5	136.3	111.4	0.090566	0.521074	0.014821	0.358848	0.295604	0.118068
	30 wks	134.9	129.6	135.8	115	0.672075	0.903073	0.077945	0.615627	0.413048	0.068008
	32 wks	134.5	119.6	123.8	113.7	0.357441	0.405112	0.201964	0.785344	0.7433	0.513348
	34 wks	134.8	101.1	113.6	101.3	0.044218	0.052658	0.046509	0.474594	0.994097	0.486105
	36 wks	122.3	111.9	103.1	120.8	0.498464	0.05897	0.91567	0.439922	0.551454	0.044838
	38 wks	123.8	113.3	99.64	132.2	0.554996	0.0983	0.608407	0.495111	0.404082	0.098399

40 wks	123.3	124.1	109.6	107.8	0.939166	0.128765	0.271931	0.211893	0.381162	0.9022
42 wks	114.2	131.6	99.36	105.8	0.237267	0.270074	0.441243	0.157656	0.201159	0.730512
44 wks	116.5	110.3	99.43	121.3	0.652898	0.112518	0.783873	0.351384	0.597984	0.171098
46 wks	115.5	117.7	90.21	124.7	0.912445	0.09135	0.629237	0.19158	0.804397	0.097603
48 wks	111.2	94	96.08	95.83	0.19321	0.316082	0.313085	0.913324	0.915496	0.990912
50 wks	102.2	96.57	76	127.5	0.756409	0.302007	0.199913	0.42958	0.092114	0.080298
52 wks	101.8	110.6	75.4	107.7	0.403834	0.075369	0.639276	0.080506	0.857123	0.151311

Gripstrength Females	Means Non-Tg	Means UBQ1	Means P497S	Means UBQ1/P497S	P values Non-Tg vs UBQ1	P values Non-Tg vs P497S	P values Non-Tg vs UB1/P497S	P values UBQ1 vs P497S	P values UBQ1 vs UB1/P497S	P values P497S vs UB1/P497S
6 wks	541.3	564.1	414.9	391.5	0.648694	0.018006	0.06625	0.005369	0.025785	0.761974
8 wks	599.5	546.6	533.2	482.5	0.203752	0.124995	0.064723	0.766687	0.30871	0.445176
10 wks	634	636.9	535.5	514.8	0.944178	0.024569	0.043689	0.027772	0.045548	0.720297
12 wks	619	638.7	510.2	566.6	0.661993	0.026977	0.247053	0.044913	0.297972	0.442372
14 wks	616.5	662.8	616.5	616.7	0.290321	0.999613	0.997263	0.231226	0.417617	0.997138
16 wks	605.6	581.4	499.8	574.5	0.59375	0.052688	0.601317	0.162387	0.908088	0.335915
18 wks	606.9	604.6	511.9	654.5	0.962483	0.067021	0.421746	0.119197	0.458011	0.044115
20 wks	666	656.3	525.1	606.4	0.804103	0.00091	0.219773	0.007763	0.36324	0.151664
22 wks	621	625.6	586.4	527	0.926012	0.522665	0.145047	0.495376	0.115794	0.412852
24 wks	675.6	663	652.2	685.9	0.757265	0.575625	0.856377	0.822021	0.730964	0.623247
26 wks	666.3	666.3	611.3	561.6	0.99918	0.175571	0.036546	0.240718	0.072026	0.464253
28 wks	629.7	661.7	528.1	632.1	0.371582	0.044744	0.964916	0.011469	0.566993	0.204275
30 wks	646.9	697.4	577.3	566.6	0.172404	0.06832	0.159553	0.00359	0.0289	0.852952
32 wks	636.5	658.7	592.4	571.7	0.625315	0.365007	0.328814	0.243536	0.258204	0.801695
34 wks	714.7	624.4	573.6	563	0.060794	0.018721	0.011766	0.498854	0.436624	0.918361
36 wks	657.2	682.8	542.6	567.8	0.561127	0.023075	0.150085	0.017865	0.113014	0.750521
38 wks	661	608.6	664.5	586.8	0.231424	0.945716	0.255072	0.286764	0.713575	0.291142
40 wks	704.5	703.2	588.5	605.3	0.976634	0.031339	0.130124	0.064942	0.181742	0.838982
42 wks	703.4	654	611	597.2	0.224326	0.045566	0.06084	0.461642	0.445167	0.862985
44 wks	713.8	668.1	652.6	578.8	0.361803	0.222441	0.05373	0.799525	0.293284	0.374764
46 wks	700.8	669	641	577.7	0.429138	0.172057	0.02824	0.606629	0.189188	0.395645
48 wks	711.6	721.1	529.9	545.8	0.798549	0.000175	0.003728	0.001326	0.01417	0.842906
50 wks	755.3	718.9	564.6	545.8	0.376297	0.00004	0.003576	0.001604	0.031722	0.799451
52 wks	722.7	678.6	603.6	479.3	0.246522	0.023655	0.000057	0.212331	0.003133	0.155022

Gripstrength Males	Means Non-Tg	Means UBQ1	Means P497S	Means UBQ1/P497S	P values Non-Tg vs UBQ1	P values Non-Tg vs P497S	P values Non-Tg vs UB1/P497S	P values UBQ1 vs P497S	P values UBQ1 vs UB1/P497S	P values P497S vs UB1/P497S
6 wks	657.3	557.3	498.3	449.3	0.007408	0.00079	0.000805	0.263924	0.130755	0.570303
8 wks	639.8	676.5	614.3	533.6	0.292623	0.491032	0.053832	0.120146	0.017291	0.189258
10 wks	686.9	710.5	638	585.8	0.492561	0.222053	0.073703	0.067788	0.021839	0.409846
12 wks	706.8	677.8	653.7	624.5	0.400398	0.199324	0.124771	0.539722	0.282847	0.628803
14 wks	713.2	691.4	657.8	670.1	0.592944	0.112068	0.399272	0.469656	0.766167	0.831605
16 wks	725.5	674.6	678.2	661.1	0.193232	0.176827	0.239581	0.929903	0.835608	0.758881
18 wks	669.4	649	701.5	684.8	0.569453	0.368935	0.762257	0.162428	0.499286	0.741872
20 wks	707.5	643.8	648.7	678.8	0.085677	0.045569	0.5813	0.893545	0.590077	0.558786
22 wks	715.1	677.9	609.9	653.9	0.287202	0.020078	0.206226	0.131433	0.594411	0.507089
24 wks	676.1	717.8	627.7	699.4	0.175073	0.125636	0.567261	0.017638	0.713195	0.171068
26 wks	724.5	718.5	701.6	661.4	0.874624	0.532122	0.14827	0.698924	0.280738	0.42193
28 wks	755	691.2	682.6	699.5	0.098275	0.10004	0.241294	0.866636	0.870292	0.796067
30 wks	691.2	681.4	703.8	671	0.785646	0.684701	0.62997	0.565985	0.844716	0.42881
32 wks	716.6	710.9	720.6	667.8	0.861362	0.889493	0.296115	0.756922	0.392843	0.226227
34 wks	791	696.2	651.4	680.4	0.048374	0.005547	0.013433	0.510602	0.82544	0.686891
36 wks	742.4	702.9	682	730.1	0.285783	0.133394	0.78475	0.654703	0.594209	0.425706
38 wks	810.4	691.4	634.4	671.3	0.001972	0.000034	0.00202	0.311007	0.77731	0.625343
40 wks	727.3	698.9	652.1	664.1	0.51106	0.030605	0.191106	0.293967	0.587095	0.78588
42 wks	759.6	776.3	643.8	671.3	0.589079	0.017723	0.078956	0.019298	0.046114	0.730071
44 wks	776.1	774.4	635.8	685.6	0.95109	0.000307	0.035688	0.001916	0.067881	0.432666
46 wks	769.4	762.5	597.2	691.1	0.829353	0.000065	0.112784	0.001197	0.230008	0.196981
48 wks	775.2	750.9	640.4	680.3	0.437625	0.001722	0.032464	0.02509	0.162227	0.560757
50 wks	742.9	738.7	597.4	700.1	0.90895	0.005038	0.368536	0.013837	0.447695	0.150791
52 wks	763.5	736.8	617.9	678.9	0.527953	0.006874	0.091841	0.078765	0.382129	0.446412

P<0.05 is highlighted in yellow

Table 2: Means of the frequency and P value comparison of the L4 axon sizes in the different genotypes

L4 Females	Bins	Means Non-Tg	Means UBQ1	Means P497S	Means UBQ1/P497S	P values Non-Tg vs UBQ1	P values Non-Tg vs P497S	P values Non-Tg vs UB1/P497S	P values UBQ1 vs P497S	P values UBQ1 vs UB1/P497S	P values P497S vs UB1/P497S
	0	0	0	0	0						
	5	2.333	19.33	52	21.33	0.150267	0.003775	0.001286	0.042561	0.847875	0.038221
	10	41.67	86.67	106.3	105	0.065176	0.006614	0.000043	0.370844	0.367418	0.944329
	15	44.33	60	56.71	77.67	0.058848	0.471999	0.01881	0.845104	0.093754	0.24552
	20	27	26.33	25.43	44.67	0.928544	0.834625	0.125794	0.902513	0.107414	0.042497
	25	18.33	19.33	18.86	23.67	0.852837	0.933057	0.508985	0.934241	0.528707	0.476779
	30	13	15.33	19.71	22.67	0.449855	0.319884	0.30024	0.496718	0.396988	0.710014
	35	11	12.67	20.57	25	0.673011	0.209323	0.14329	0.280135	0.162726	0.592029
	40	9.333	16.33	19.86	20.33	0.122985	0.077934	0.222862	0.542461	0.653508	0.946258
	45	12	17	22.29	31.33	0.138571	0.129795	0.185371	0.407941	0.299896	0.358655
	50	16	21	21.14	19	0.352882	0.392872	0.666276	0.979437	0.740233	0.72895
	55	13.33	26	23	23	0.026965	0.005765	0.231797	0.393597	0.716776	>0.999999
	60	16	30.33	22.57	25	0.041544	0.205331	0.166136	0.18223	0.465395	0.669139
	65	18.33	30.67	20.57	20.67	0.292215	0.589473	0.664339	0.183181	0.403658	0.983491
	70	23.67	33.67	21.43	20.33	0.122558	0.214894	0.369564	0.007004	0.089009	0.66904
	75	21	34	19.86	26.33	0.235092	0.736588	0.303548	0.048996	0.474302	0.116484
	80	29.33	24.67	18.43	21.33	0.448352	0.045414	0.204379	0.212617	0.562184	0.535606
	85	28.33	26.67	17.86	21	0.769407	0.0704	0.275589	0.059799	0.122952	0.481722
	90	31	25.67	14.43	14	0.519288	0.004897	0.0422	0.026944	0.099315	0.888655
	95	32	20.33	11.57	15.33	0.009176	0.000053	0.053447	0.007141	0.446944	0.391977
	100	31.67	10	8.714	9.667	0.00289	0.000012	0.012139	0.579839	0.948049	0.772333
	105	27	16.33	5.429	8.667	0.021379	0.000009	0.00195	0.000991	0.037166	0.141961
	110	15.33	11.67	6.429	7.667	0.57695	0.015611	0.176622	0.099011	0.423997	0.468071
	115	21.33	9.333	4.714	6.667	0.090627	0.000301	0.024886	0.195994	0.632124	0.467552
	120	16	4.333	4.143	6.667	0.04781	0.001289	0.082794	0.919134	0.477587	0.187515
	125	17	7	3.429	6	0.177048	0.000116	0.049864	0.343846	0.882512	0.277216
	130	8.333	4.333	1.429	4.667	0.292144	0.002454	0.234932	0.10482	0.905069	0.014761
	135	8.333	1.333	2.429	2.333	0.126531	0.022975	0.17851	0.299588	0.638768	0.929691
	140	6	2	1.571	3	0.306569	0.055497	0.40352	0.727646	0.613011	0.177898
	145	5.333	3.667	1.286	3.667	0.658429	0.004544	0.462807	0.264326	>0.999999	0.050933
	150	4.667	2.333	1.571	2	0.390739	0.013919	0.116117	0.658758	0.904299	0.716
	155	3	2.667	0.7143	3	0.859308	0.008858	>0.999999	0.127084	0.889917	0.066258
	160	3	2.667	0.7143	1.667	0.908657	0.003836	0.373901	0.272397	0.749647	0.294236

L4 Males	Bins	Means Non-Tg	Means UBQ1	Means P497S	Means UBQ1/P497S	P values Non-Tg vs UBQ1	P values Non-Tg vs P497S	P values Non-Tg vs UB1/P497S	P values UBQ1 vs P497S	P values UBQ1 vs UB1/P497S	P values P497S vs UB1/P497S
	0	0	0	0	0						
	5	10	39	33.33	25.67	0.150405	0.268188	0.161482	0.8231	0.495851	0.714362
	10	71.33	106.7	53	118	0.302901	0.586701	0.16736	0.111447	0.638204	0.052544
	15	59	48.33	38.33	85.67	0.334769	0.179521	0.124744	0.383463	0.031473	0.028476
	20	34.67	22.67	23.33	37	0.139677	0.167209	0.748436	0.741521	0.002445	0.006862
	25	19	12.67	20.67	24.67	0.233907	0.731102	0.210224	0.117955	0.019891	0.279083
	30	12.67	9.333	18.33	22	0.262865	0.21587	0.014513	0.113747	0.016354	0.441348
	35	6.333	6.667	20.33	27.67	0.900148	0.049431	0.003291	0.037494	0.00109	0.21802
	40	11	12	17.67	29.67	0.629095	0.214384	0.0001	0.288222	0.00035	0.052065
	45	13.67	8.333	22.33	33.33	0.364474	0.268454	0.049125	0.047575	0.009459	0.183458
	50	16	8	19	32.67	0.076291	0.665047	0.010905	0.12373	0.000305	0.080371
	55	12.67	13.33	19.67	30.33	0.916365	0.367478	0.045464	0.327045	0.023178	0.143784
	60	15	17	17	21.67	0.71826	0.760854	0.189004	>0.999999	0.245492	0.383537
	65	19	21	23	21.67	0.841301	0.68957	0.799416	0.555073	0.887147	0.771715
	70	19.33	25.67	18.67	21.33	0.485966	0.929105	0.787646	0.267335	0.460461	0.436975
	75	28.33	24.67	19.67	13.33	0.798637	0.544719	0.313919	0.278008	0.038363	0.045728
	80	26.33	23.67	13.67	16	0.787038	0.226983	0.313449	0.023579	0.067146	0.256435
	85	33	19.33	13	14	0.113507	0.05198	0.046229	0.155181	0.058163	0.784257
	90	35	23.67	8	9.333	0.110583	0.006824	0.007706	0.004936	0.005467	0.530412
	95	24.33	19	4.667	13.67	0.064677	0.000771	0.073388	0.0044	0.310293	0.122443
	100	32.67	19	9.333	7.333	0.06148	0.009468	0.005331	0.065733	0.024896	0.515752
	105	21.67	17.33	5.667	8.333	0.667942	0.000876	0.011056	0.274142	0.397819	0.333516
	110	21	15	5	6.667	0.369889	0.015024	0.040678	0.114346	0.217294	0.661225
	115	18	16.33	6.667	6.333	0.844882	0.054775	0.04515	0.238988	0.220636	0.802482
	120	16.33	8.667	4	2	0.208696	0.032641	0.018124	0.27206	0.131778	0.116117
	125	11.33	10.67	2	3	0.889319	0.042592	0.058755	0.052685	0.073471	
	130	16	4	2.333	3.333	0.020243	0.006862	0.008971	0.488251	0.77558	0.467605
	135	12.67	3.667	2	2	0.034892	0.021379	0.021379	0.066767	0.066767	>0.999999
	140	5.333	4.667	1.667	2	0.802482	0.08352	0.099982	0.232566	0.274577	0.724659
	145	9	3.333	1.667	0.6667	0.077877	0.037997	0.023346	0.151835	0.023215	0.250815
	150	4.667	2.333	2	3	0.364956	0.224122	0.457253	0.841596	0.724659	0.435331
	155	4.667	2.667	1.333	0.6667	0.416866	0.180077	0.134196	0.2302	0.144704	0.421648
	160	4.333	1	0	2	0.131778	0.069934	0.277069		0.158302	0.025721

P<0.05 is highlighted in yellow

A

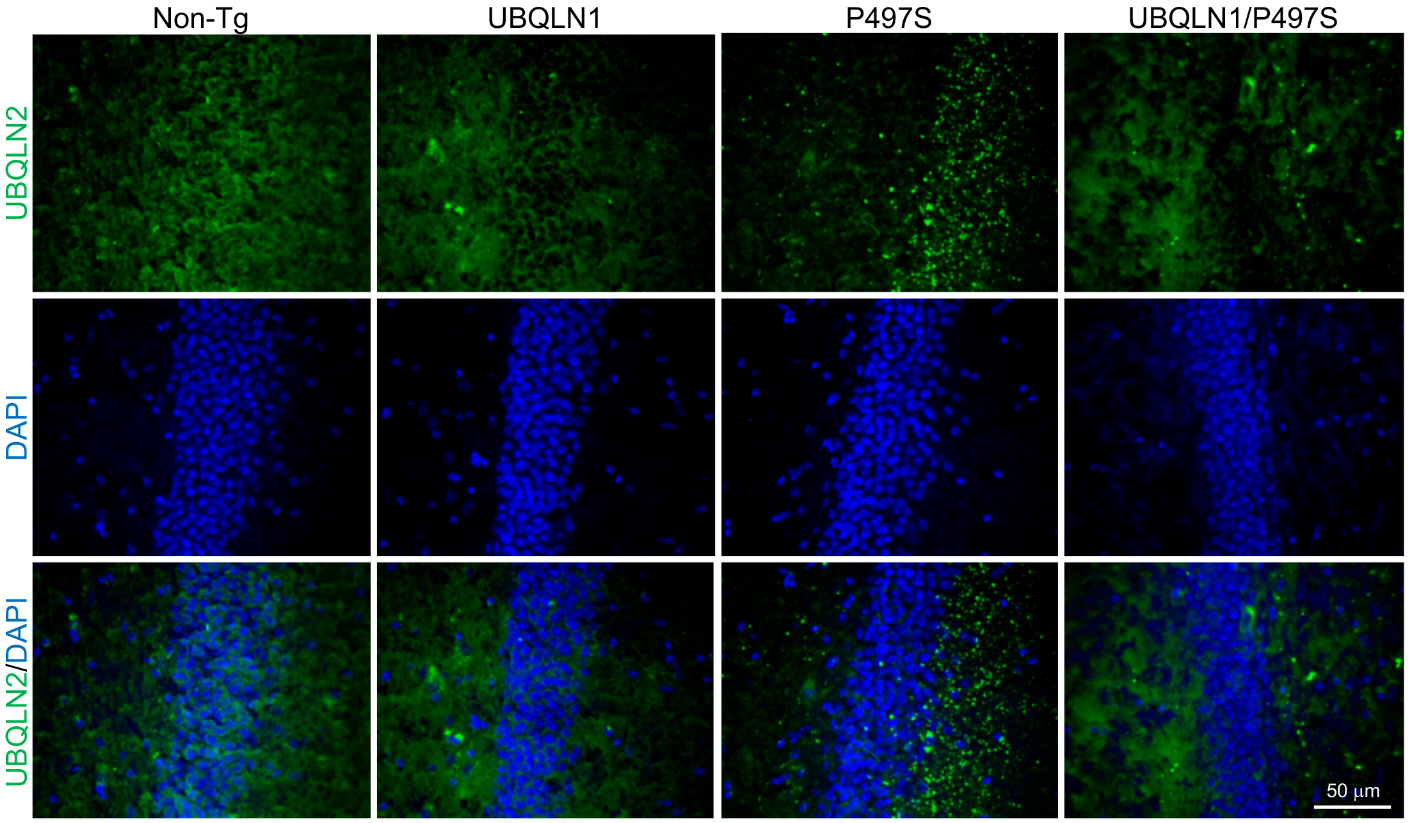

Non-Tg
UBQLN1
P497S
UBQLN1/P497S
UBQLN2
DAPI
UBQLN2/DAPI
50 µm

B

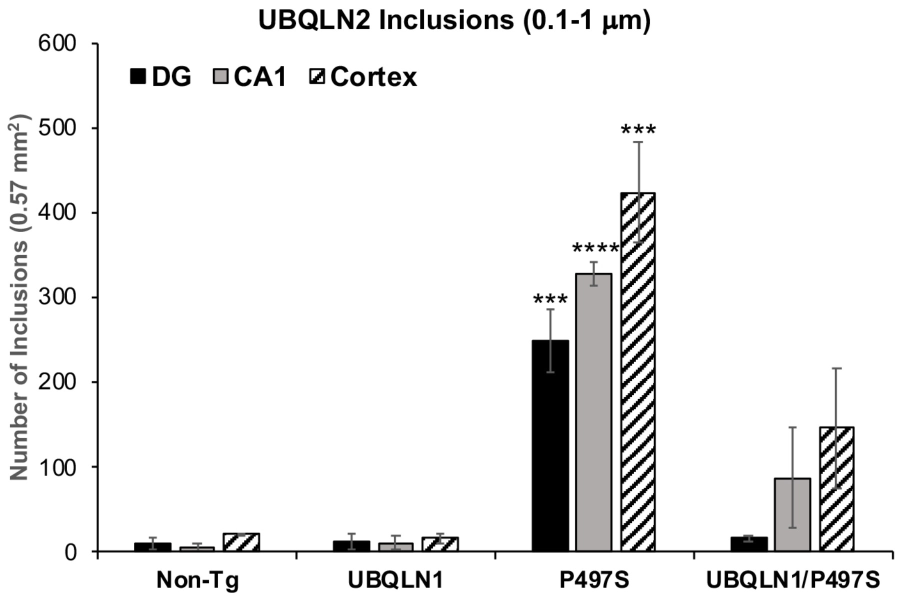

UBQLN2 Inclusions (0.1-1 µm)
DG
CA1
Cortex
Number of Inclusions (0.57 mm²)
600
500
400
300
200
100
0
***
****
***
Non-Tg
UBQLN1
P497S
UBQLN1/P497S

Fig S1

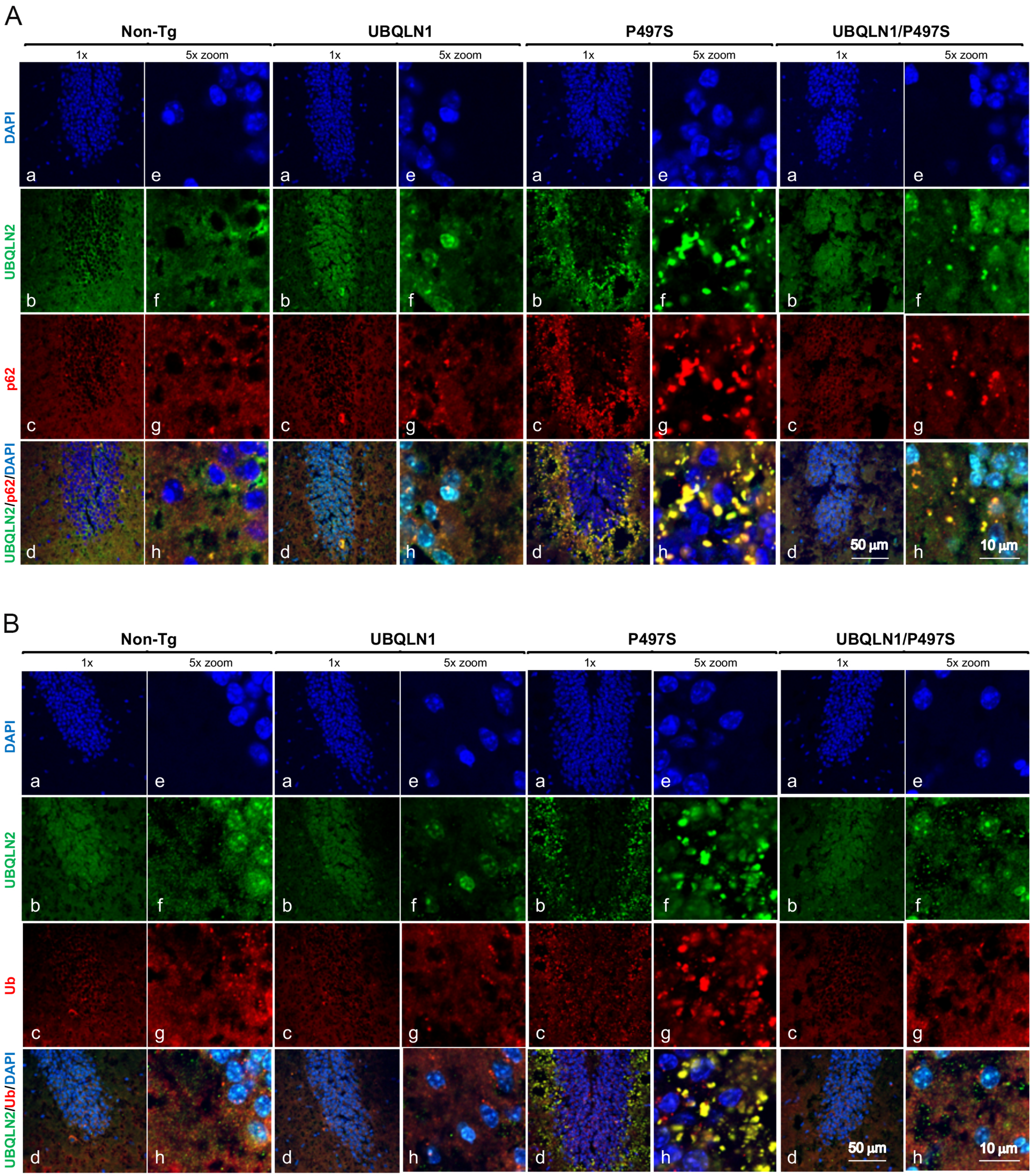
A
Non-Tg
UBQLN1
P497S
UBQLN1/P497S
1x
5x zoom
DAPI
UBQLN2
p62
UBQLN2/p62/DAPI
a
b
c
d
e
f
g
h
50 µm
10 µm
B
Non-Tg
UBQLN1
P497S
UBQLN1/P497S
1x
5x zoom
DAPI
UBQLN2
Ub
UBQLN2/Ub/DAPI
50 µm
10 µm

Fig S2

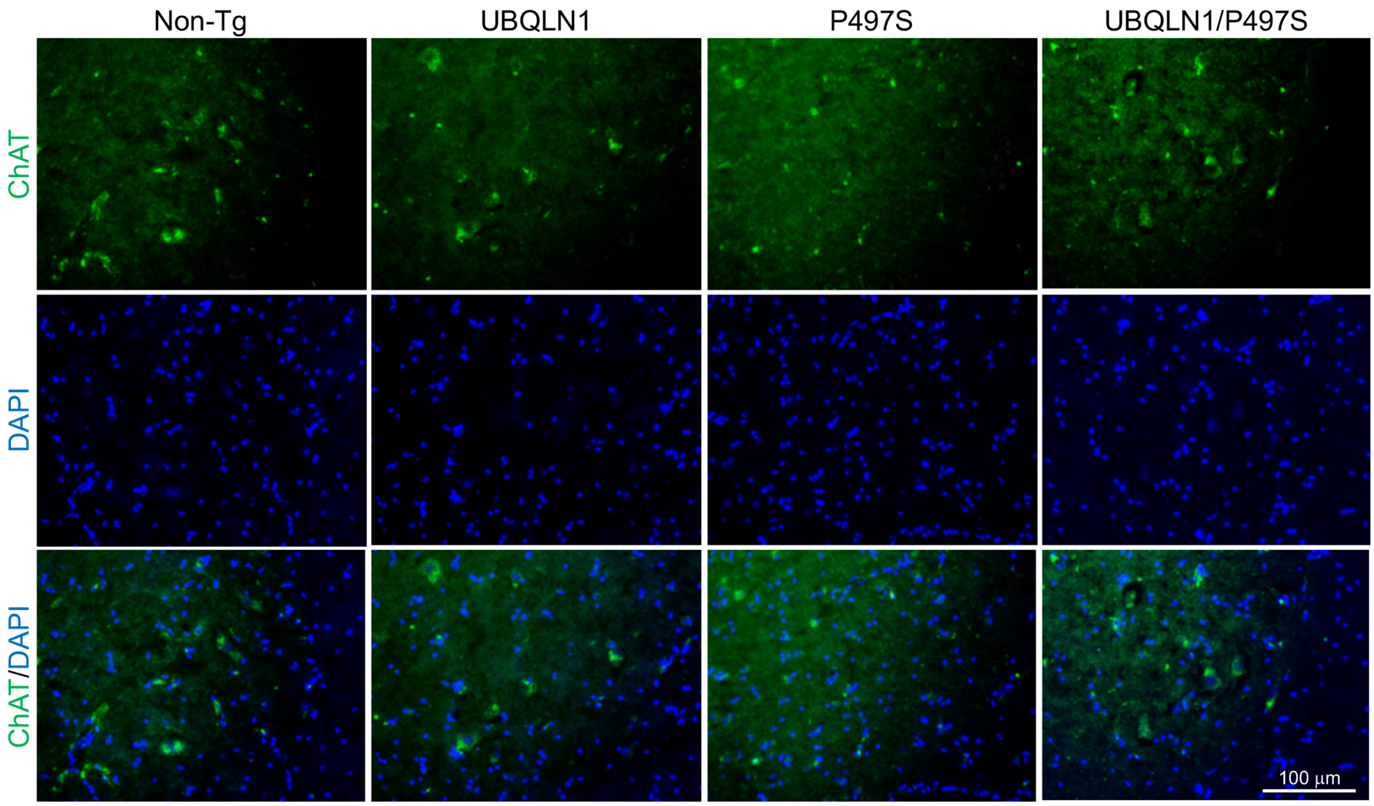

Fig S3

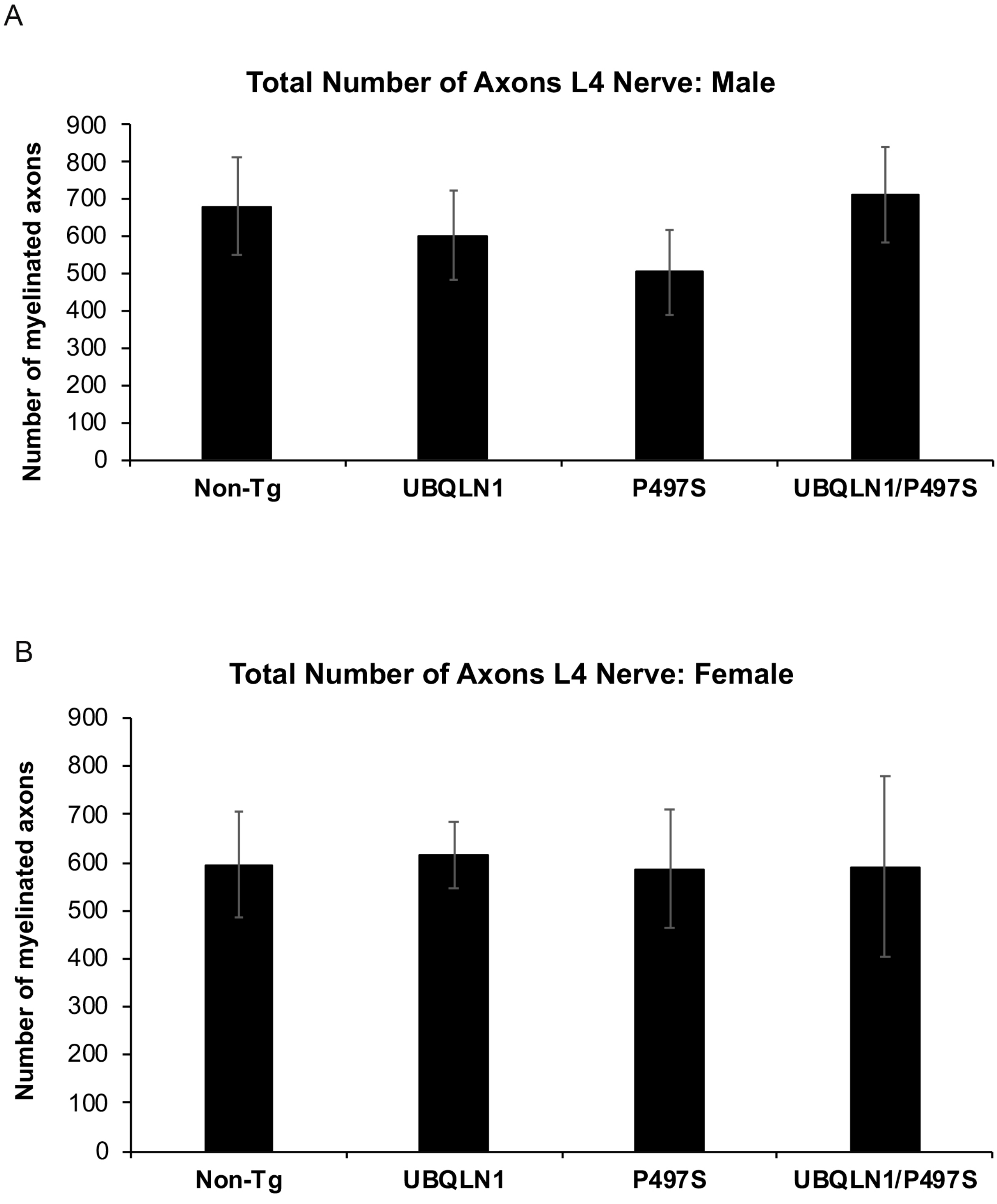
A
Total Number of Axons L4 Nerve: Male
Number of myelinated axons
900
800
700
600
500
400
300
200
100
0
Non-Tg
UBQLN1
P497S
UBQLN1/P497S
B
Total Number of Axons L4 Nerve: Female
Number of myelinated axons
900
800
700
600
500
400
300
200
100
0
Non-Tg
UBQLN1
P497S
UBQLN1/P497S

Fig S4
